# Inferring Gene Regulatory Networks from RNA-seq Data Using Kernel Classification

**DOI:** 10.3390/biology12040518

**Published:** 2023-03-29

**Authors:** Amira Al-Aamri, Andrzej S. Kudlicki, Maher Maalouf, Kamal Taha, Dirar Homouz

**Affiliations:** 1 Department of Physics, Khalifa University of Science and Technology, Abu Dhabi P.O. Box 127788, United Arab Emirates; 2 Department of Biochemistry and Molecular Biology, Institute for Translational Sciences, The University of Texas Medical Branch, Galveston, TX 77555, USA; 3 Department of Industrial and Systems Engineering, Khalifa University, Abu Dhabi P.O. Box 127788, United Arab Emirates; 4 Department of Electrical and Computer Science, Khalifa University, Abu Dhabi P.O. Box 127788, United Arab Emirates

**Keywords:** bioinformatics, RNA-seq, microarray, regulatory networks, kernel classification, gene expression profiling

## Abstract

**Simple Summary:**

Understanding the relationships between genes is crucial to identify the genetic program of organisms. This research could potentially have implications for this understanding. This study aims to build a genetic network for the yeast genome by recognizing interacting genes. This is performed by incorporating different mathematical methodologies and classification techniques to predict relations between two genes. High accuracy is achieved across several tests to identify network interactions. Our findings provide new insights and potential interactions between genes in the yeast genome network. Our results are highly relevant to the field, as they emphasize the power of classification and its role in improving the current understanding of the yeast genome network.

**Abstract:**

Gene expression profiling is one of the most recognized techniques for inferring gene regulators and their potential targets in gene regulatory networks (GRN). The purpose of this study is to build a regulatory network for the budding yeast *Saccharomyces cerevisiae* genome by incorporating the use of RNA-seq and microarray data represented by a wide range of experimental conditions. We introduce a pipeline for data analysis, data preparation, and training models. Several kernel classification models; including one-class, two-class, and rare event classification methods, are used to categorize genes. We test the impact of the normalization techniques on the overall performance of RNA-seq. Our findings provide new insights into the interactions between genes in the yeast regulatory network. The conclusions of our study have significant importance since they highlight the effectiveness of classification and its contribution towards enhancing the present comprehension of the yeast regulatory network. When assessed, our pipeline demonstrates strong performance across different statistical metrics, such as a 99% recall rate and a 98% AUC score.

## 1. Introduction

Reconstructing the causal relationships in genetic networks, including regulatory interactions, is important for understanding biological processes and disease mechanisms. Computational prediction of regulations from transcriptomics data can be useful for selecting and prioritizing candidate genes that can serve as biomarkers or potential drug targets. The two main technologies widely used for transcriptome-wide gene expression profiling are microarrays and next-generation RNA sequencing (RNA-seq). Here, we discuss an approach to reconstructing causal networks based on the analysis of transcription data from microarray and RNA-seq. While microarrays have been the preferred method due to cost-effectiveness and ease of data analysis [1,2,3], RNA-seq has advantages in sensitivity, coverage of differentially expressed genes, and identification of isoforms and non-coding genes [4,5,6,7]. An in-depth review summary of the differences between RNA-seq and microarray approaches is included in the Supplementary Materials.

Prediction analysis is one of the compelling comparative methods used to identify the pros and cons of using RNA-seq and microarray-based models. Several studies show the effects of these data models on different organisms and various classification systems. A prediction analysis study for identifying cancer biomarkers presented a comparison between RNA-seq and microarray-based classifiers to identify the significance of each in the field of clinical practice [8]. Other studies assess the prediction accuracy for data derived from RNA-seq and microarray [9,10]. Their results show an interesting view of the potential and the advantages of using each profiling technique. They conclude that conducting a comparison to point out which technique outperforms the other is not the optimal approach. A favored method is understanding how each profiling technique should be utilized in different conditions to maximize the results.

In this work, we adopt a methodical approach to evaluate and compare the performance of models with various classifiers for regulatory network prediction using either RNA-seq or microarray-based input data. In our previous work, we detected regulatory associations for *Saccharomyces cerevisiae* genes using microarray expression data derived from various experimental settings [11]. In the present article, we extend our work to analyze and classify RNA-seq data for the *S. cerevisiae* genome as well. Each dataset (RNA-seq and microarray) represents a variety of experimental conditions to capture the nature of data and extract the prediction features. We will study the difference in prediction accuracy between microarray data and RNA-seq data. We will shed the light on the performance of these datasets across kernel classification models; one-class, two-class, and rare-event classifiers. Finally, we will study the impact of RNA-seq data normalization techniques on the prediction accuracy of *S. cerevisiae* gene pairs.

The practical strength of our approach is in improving the target selection process, making informed decisions on which genes or gene products are the most promising targets of experimental studies, and prioritizing the use of laboratory resources when only a limited number of genes can be tested experimentally. The approach can be used in model organisms (such as *S. cerevisiae*) to study conserved regulatory relationships, or in human or human pathogens to identify drug targets or biomarkers. Our findings provide new insights into the interactions between genes in the yeast regulatory network. We have also identified new potential targets for transcription factors using both profiling technologies by inferring the network interactions. Our results emphasize the power of classification and its role to improve the current understanding of the yeast regulatory network.

The rest of the article is structured as follows. Section 2 presents the analysis pipeline of our proposed method. It covers RNA-seq data analysis, data preparations, and training, and introduces the kernel classifiers used in this work. We discuss the experimental results in Section 3. Finally, we provide conclusions in Section 4.

## 2. Methods

**Figure 1:**
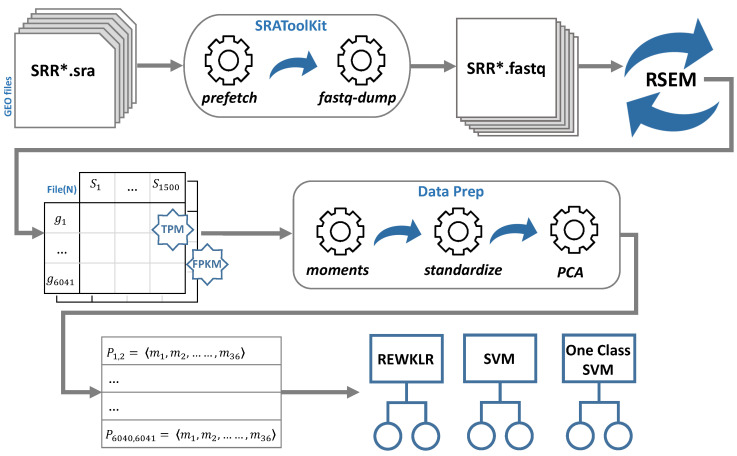
The general pipeline adopted in this work is represented in three major sections. Section 2.1: RNA-seq data analysis is summarized in the top part of the figure. Sequence Read Archive files are converted to fastQ files using SRAToolKit, then fed to the RSEM tool for gene quantification. The middle part illustrates the data preparation for system classifications, Section 2.2. The files containing the values of count reads for the matrix (genes x samples) are prepared for analysis by calculating the moments for each pair of genes, standardizing each moment, and then applying the Principal Component Analysis (PCA). The bottom part shows Section 2.3: training data and models. All pairs of genes are represented by 36 moments and fed to the kernel classification models for training and testing.

In this section, we present the methods adopted for the analysis of RNA-seq data. We have followed a similar approach to our previous study targeting microarray data. For an in-depth understanding of the microarray analysis method adopted readers may refer to Alaamri et al. [11]. The pipeline introduced in this section is divided into three main sections: RNA-seq data analysis (Section 2.1), data preparation for system classifications (Section 2.2), and training data and models (Section 2.3). An overall presentation of the customized pipeline followed in this work is illustrated in Figure 1.

### 2.1. RNA-seq Data Analysis

The goal of this section is to create a list of genes each represented by normalized reads from different experiment conditions. The approach follows a typical RNA-seq workflow for differential expression (DE) analysis. The process of data collection is automated as a pipeline using the steps and tools listed below:

- GEO database: The Gene Expression Omnibus (GEO) was used to obtain the *S. cerevisiae* RNA-seq expression data [12]. We used three main GEO platforms to download RNA-seq records (GPL19756, GPL17342, and GPL13821). Each of these platform files contains different experimentation conditions (series) and various samples for each series. To obtain these samples and specifically those representing RNA-seq data delivered by using Illumina sequencing technology, we used the BioProject ID that links the GEO sample to the correspondent Sequence Read Archive (SRA) accession IDs [13]. We randomly selected and downloaded around 1500 SRA accession IDs.
- SRA-Toolkit: This is another well-designed service by NCBI. SRA-Toolkit is a collection of tools to utilize SRA data [14]. We mainly used two operations; namely *prefetch* and *fastq*-*dump*. *Prefetch* was used to download sequence files in the compressed SRA format using the SRA accession IDs obtained from the previous step. These sequence files were then used by the *fastq*-*dump* sub-tool to retrieve the SRA fastQ files. This process required a significant amount of time and disk space. At this point, all the files were prepared for mapping and transcript quantification to obtain the reads for each gene.
- RSEM tool: Finally, the fastQ files were fed to the RNA-seq by Expectation Maximization (RSEM) package tool [15]. RSEM is an open-source software tool for gene quantification using single-end or paired-end RNA-seq data. It also utilizes Bowtie; a powerful and efficient alignment software [16]. We used the main functions showcased in the typical RSEM workflow: *rsem*-*prepare*-*reference* and *rsem*-*calculate*-*expression*.
  The reference was prepared by obtaining the yeast FASTA-formatted file from Ensembl genome browser release 82 [17]. Then, RSEM takes as input the fastQ files downloaded previously using SRA-Toolkit. The tool maps each fastQ file and outputs the calculated expression as gene read counts. The read counts are normalized by default with Transcripts Per Kilobase Million (TPM) and Fragments Per Kilobase Million (FPKM) normalization metrics. In Section 3, we showcase the effect of the normalization method on the prediction accuracy for the gene–gene association. For each normalization metric, we merged all the read counts according to their gene correspondence to create a matrix table of genes (rows) and sample counts (columns). The output files of this step are referred to as File(N), where N denotes the normalization method (TPM or FPKM).

It is worth noting that gene names are equalized to avoid any confusion between processes and to ensure data coherence. The gene names for all genes in File(N) and in the databases mentioned in Section 2.3 are standardized according to the UniProt Knowledgebase (UniProtKB) [18]. UniProtKB presents each gene name in a primary name, synonyms, and ordered locus name. We followed the order locus name for its uniqueness by converting all the gene names. This step upgraded the efficiency of all previous and later processes. Next, the data was prepared for classification.

### 2.2. Data Preparation for Classification

#### 2.2.1. Microarray

As previously stated, the microarray data were acquired from our previous work [11]. The data went through several steps that are mentioned in detail in Section 2.2.2. The processed data ready for training consisted of around 43 million pairs of genes each represented by 36 moments. In this paper, we rerun the training process for microarray data to keep up with the updated gene regulatory databases used for training and classification.

#### 2.2.2. RNA-seq

The output of Section 2.1: File(N), consists of 6041 genes, each defined by a vector of 1500 normalized read counts. We used this data to construct the gene regulatory network (GRN) and define each pair of genes by a vector of features. The moments ($E[x^{n}y^{m}]$) represent the features and are calculated using the joint probability distribution of all pairs of genes. It is worth noting that higher moments can provide more information to capture causal relationships because they measure higher order dependencies between random variables. In contrast to correlation-based measures that only capture linear relationships between variables, higher moments can capture nonlinear relationships and higher order interactions. These higher order interactions may be indicative of causal relationships that are not captured by simpler measures.

**Figure 2:**
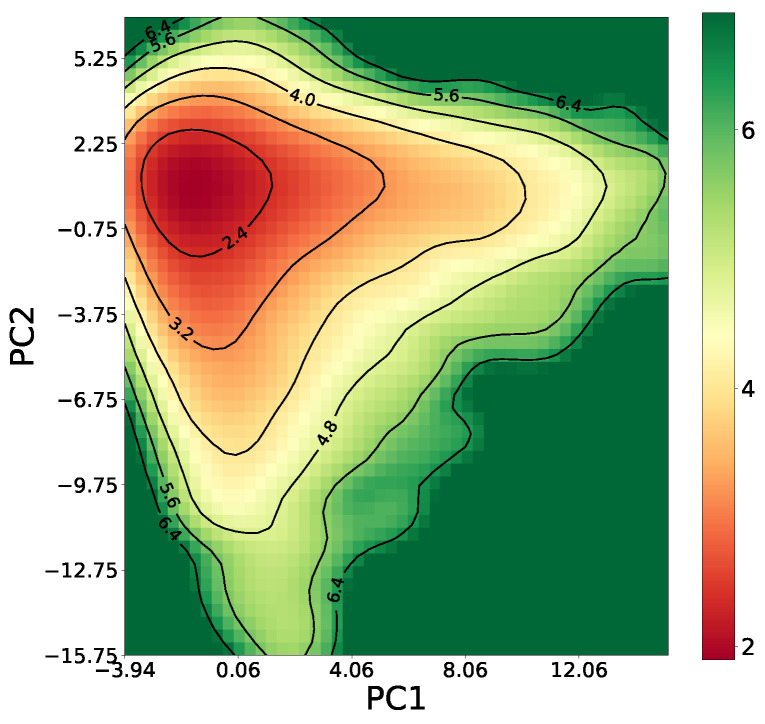
A 2D histogram is showing the structure of true positive using FPKM training data.

The outcome of this step is the data matrix that consists of 18,243,820 pairs of genes, each pair represented by a vector of 49 moments. All the moments are then standardized for all pairs of genes. We applied the Principal Component Analysis (PCA) to first convert the moments to principal components and then identify the significant moments [19]. This reduced the dimensionality of moments (principal components) to 36. We aimed to use the moment size that produced the best training accuracy which was revealed as 36 in the previous study [11]. We illustrate the structure of our training data in terms of the top principal components in Figure 2. Next, we analyzed the pairs’ vectors using different classification models (i.e., Rare Event Weighted Kernel Logistic Regression (REWKLR), and one-class and two-class Support Vector Machine (SVM).

### 2.3. Training Data and Models

The training data consisted of two classes; related and unrelated pairs to aim at constructing the undirected gene regulatory network. The GRN data for *S. cerevisiae* are found in various databases such as YeasTract [20], YTRP [21], Harbison data [22], YeastPathways [23], and KEGG pathways [24]. The list of abbreviations for these databases is enlisted at the end of the article. In this work, we use YeasTract (YT) for training. YT is a continually updated repository curated by using mainly the information from Saccharomyces Genome Database (SGD) and Gene Ontology (GO) consortium. It consists of 213,807 regulatory associations between transcription and target genes. We later use YTRP, YeastPathways (YP), and KEGG for data testing and prediction. All of the mentioned databases offer easy access to downloadable material. As mentioned previously, all the gene names in the databases were converted to their ordered locus name as found in UniProtKB. The related class in the training data consists of vectors of moments for the pair of genes found in the YT repository. This class will also be referred to as the positive class, and each pair vector is denoted as “1”.

**Figure 3:**
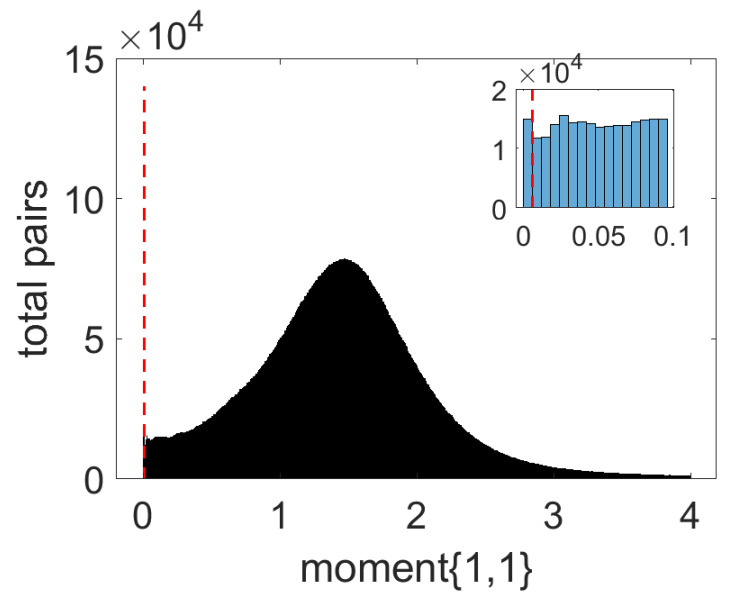
An example histogram plot for FPKM correlation data.

Determining the unrelated pairs presents a challenge to the prediction association since it is questionable to assume that any pair absent from all GRN database sources is unrelated. To tackle this, we set up the following assumption. The lowest correlated pairs of genes are to be considered in the negative class for training. Moment $E[x^{1}y^{1}]$ corresponds to the correlation between the two genes in the pair. We used the absolute values for moment {1,1} for all gene pairs to draw the histogram as demonstrated in the example shown in Figure 3. We then drew out the lowest 10,000 pairs to categorize them under unrelated pairs (negative class). Each of these pairs’ vectors is indicated with “0”. In Figure 1, the table of vectors for each pair of gene $P_{x,y}$ is shown as an example of how the training data looks upon classification.

### 2.4. Kernel Classification Methods

This section introduces the different classifiers used to identify the best fit for our data. In our previous work [11], we used the REWKLR classifier to train and predict the microarray data [25]. We aimed to employ the same mechanism for the RNA-seq data. Additionally, we train and predict both data types with SVM and one-class SVM. A summary description of each classifier is itemized below:

- REWKLR is identified as a rare-event classifier where the unrelated pairs outnumber the related ones. This captures the natural dynamics of genetic data and considers the sparsity of the data. In particular, the data in this article exhibit the genetic data nature where the percentages of zeros (unrelated pairs) exceed that of ones (related pairs). REWKLR embeds the kernel in a typical Logistic Regression model to reflect the nonlinearity of data separation. The performance of REWKLR was shown to compete with other classifiers such as SVM [25,26,27]. The parameters optimized for REWKLR are the regularization variable (λ) and the dual variable (σ).
- SVM is one of the widely used supervised learning models. It efficiently uses the kernel trick to separate two or more classes with nonlinear classification. It is also one of the highly effective models to handle a sparse number of features in a high-dimensional representation. However, it might not always be the optimal solution for capturing rare events [28]. Other methods such as REWKLR [26] and hexagonal cellular networks [29] consider the rarity of events in classification. We used the Gaussian radial basis function (RBF), a commonly used kernel with SVM, to train and predict our data. The hyperparameters optimized for training are the penalty parameter of the error term (*C*), and the Gamma parameter (γ).
- One-class SVM is shown to be the version of SVM that detects rare events more sufficiently [30,31]. To validate that, we tested our data with this model since the negative class might not be deterministic as stated previously in Section 2.3. Hence, we trained the positive class data features. We used the RBF kernel in one-class SVM as well. The parameters optimized in this model, however, are the Gamma parameter (γ) and the Nu parameter (ν) which acts both as a lower bound and an upper bound for the number of features.

At this point, there were three datasets ready for training. The first is microarray data, the second is RNA-seq data normalized by TPM, and the third is normalized by FPKM. Each training dataset consists of a matrix of 20,000 pairs and 36 features, with equal sizes of ones and zeros (positive and negative classes). As for the one-class SVM classifier, we used only the 10,000 ones that are part of the training dataset. Several tests were attempted to find the best data fit and accuracy by tuning the kernel parameters in all classifiers. Details of training accuracy and parameters for each training dataset are discussed later in Section 3.1. It is important to note that we do not follow the training parameters for microarray data used in previous work. Since the GRN databases are constantly updating, the microarray data is trained again with REWKLR to have a fair comparison among datasets.

## 3. Results and Discussion

In this section, we address the data preparation and system specifications for the workflow setup shown in Figure 1. We further compare the performance of three kernel classifiers in terms of prediction accuracy. We report the training accuracy measures and the findings in Section 3.1 and Section 3.2, respectively.

### 3.1. Data Preparation and System Specification

The pipeline unit was developed by utilizing an on-premise High-Performance Computer cluster that provides parallel job execution. It was essential particularly for the RNA-seq analysis since it required large computational power and memory. Converting each of the 1500 SRA files to fastQ and then calculating the count read from each fastQ file were parallelized. Jobs that were also run in parallel for a faster and more effective approach were calculating the moments and running the PCA analysis. The three datasets TPM, FPKM, and microarray were then fed to the classifiers. REWKLR is built on MatLab guided by [26]. SVM and one-class SVM classifiers were implemented using the open-source Python library Scikit-learn [32]. All programming platforms were installed on a system with the following specifications: Intel(R) Core i7 processor @ 3.4 GHz and 16 GB RAM, running Windows 10. All classifiers required parameter optimization by testing over a range of values and selecting the best-fitting ones. A bootstrapping sampling technique was used to iteratively select subsets of the available data for training and testing in order to find the optimal parameters that achieve the highest average accuracy for two classes in the case of binary classification, or the highest accuracy for the positive class in the case of one-class classification. Training each classification model with three datasets resulted in the parameters and accuracy measures reported in Table 1.

**Figure 4:**
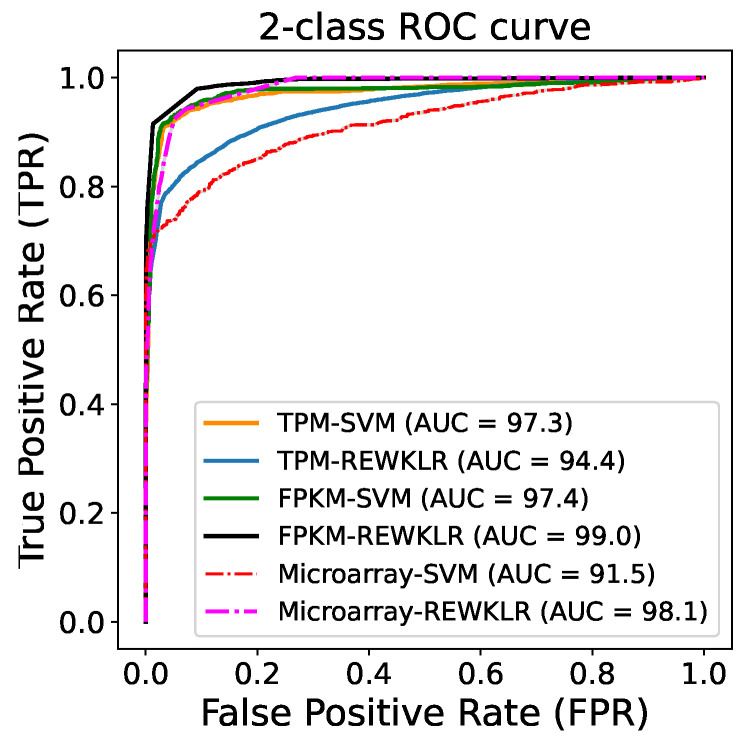
ROC curve for training data showing in general the recall is increased at a low fallout.

Overall, RNA-seq data achieves higher training accuracy over microarray. The training accuracy fluctuates across classification models for both normalization methods TPM and FPKM. We illustrate the Receiver Operating Characteristic (ROC) curve in Figure 4 to assess the quality of the training for REWKLR and SVM. One-class SVM is skipped since it does not produce a false positive rate. The curve was calculated using 500 measurements for each threshold and by resampling the training data to produce the error bar. A better view of the error bars for each dataset is included in the Supplementary Materials. Overall, all datasets showed good performance across classifiers.

### 3.2. Prediction Results

**Figure 5:**
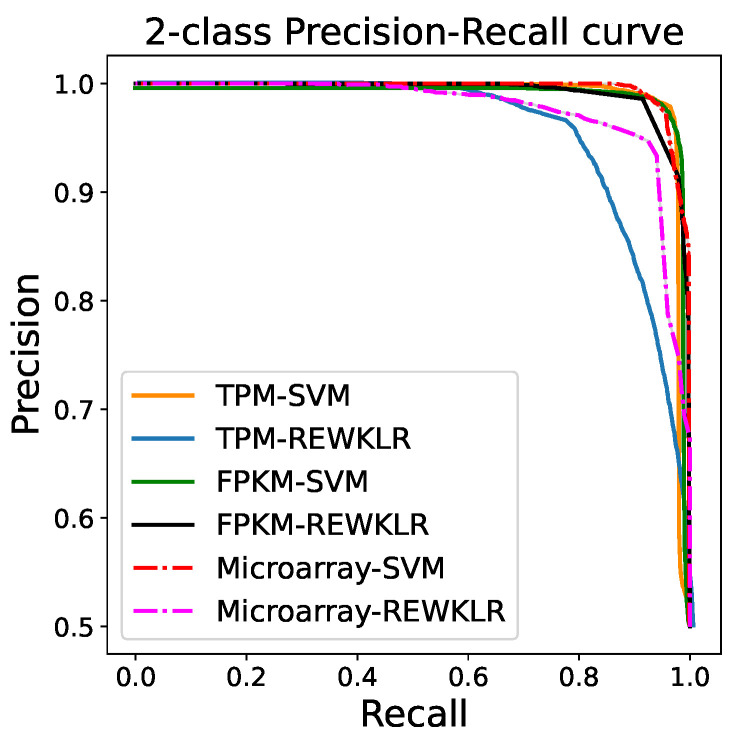
Two-class Precision–Recall curves for SVM and REWKLR. RNA-seq data have better performance in both classifiers in comparison to microarray.

The performance of each model was validated with the following databases: YeasTract (YT) [20], YTRP [21], YeastPathways (YP) [23], and KEGG pathways [24]. We downloaded a total of 390,378 gene–gene interactions from KEGG, 338,312 gene–gene interactions from YeastPathways, and 41,817 regulatory interactions from YTRP, from which randomly selected datasets were utilized for testing. In total, we had twelve different testing datasets for all benchmarks. There were three YT datasets for TPM, FPKM, and microarray data and for YTRP, YP, and KEGG. To avoid training bias, the datasets were randomly picked by excluding the YT pairs used for training. We did not mix the datasets into one dataset but rather test them separately. In particular, each testing dataset consisted of 10-20 thousand vectors of pairs. We evaluated the accuracy of the three classification models in terms of class accuracy as shown previously in Table 1, and in terms of True Positive Rate (TPR), and False Positive Rate (FPR) as was shown in Figure 4. We further computed the recall, and precision to evaluate the accuracy of identifying related genes. The Precision–Recall (PR) curve is shown in Figure 5 to emphasize the performance balance across several thresholds for the testing data. The curve was calculated using 500 measurements for each threshold and by resampling the testing data to produce the error bar. A better view of the error bars for each dataset is included in the Supplementary Materials.

**Figure 6:**
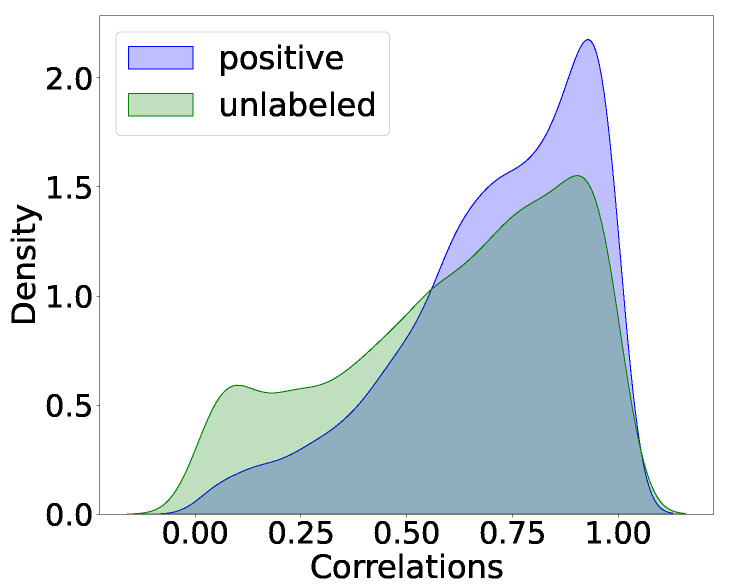
Density plots for correlations for positive and unlabeled interactions.

To illustrate the complexity of the relationships predicted, we added density plots to compare the distributions of correlation values between linked and unlinked pairs. Figure 6 displays both plots overlapped, indicating that relying solely on correlation is insufficient to differentiate between connected and unconnected genes.

**Figure 7:**
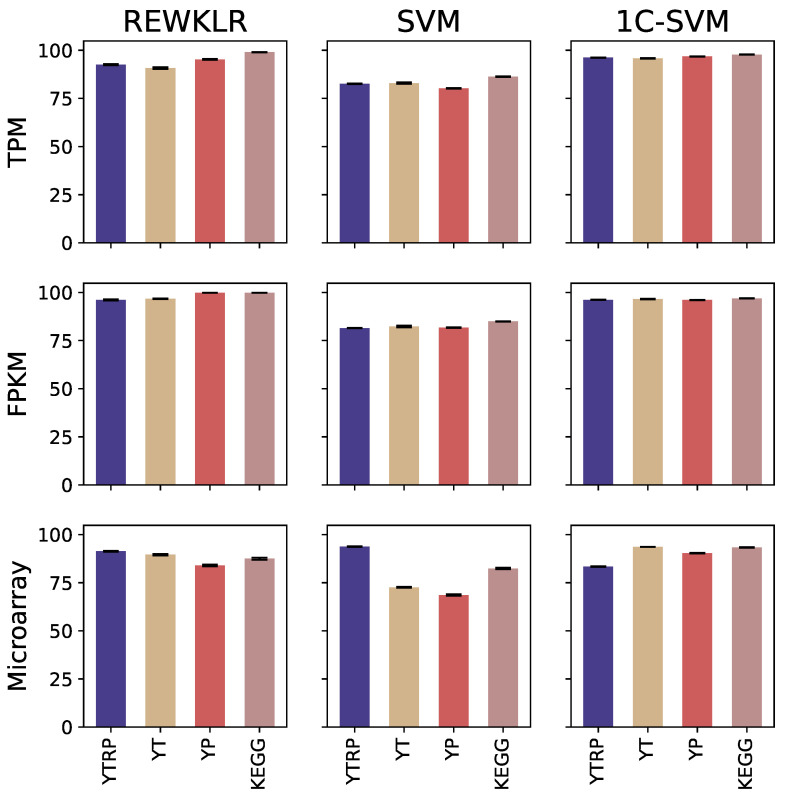
The performance results reported for the combination of all datasets, benchmarks, and classification models in terms of average recall. This resulted in curating twelve different testing datasets used for prediction. Each dataset (RNA-seq TPM normalized, RNA-seq FPKM normalized, and microarray) was validated using four different benchmarks (YTRP, YT, YP, and KEGG). The curated datasets were classified and evaluated using classifiers (REWKLR, SVM, and one-class SVM).

Next, we chose the recall accuracy measure to display the performance of all classifiers. The bar plot in Figure 7 demonstrates the performance of each classifier using different testing datasets over several measurements. The Supplementary Materials include a different version of the figure by focusing on the range 70–100% to provide a better view of the error bars. The main observation is that all classifiers performed well in terms of recall across all datasets. This emphasizes the power of classification in identifying yeast GRN using RNA-seq-based and microarray-based datasets. In particular, we see that REWKLR and one-class SVM scored the highest recall on average across datasets. In addition, REWKLR and one-class SVM performed similarly in most datasets, which indicates the classifiers’ success in capturing rare events more sufficiently by recognizing the sparsity of the data. Another particular observation is towards one-class SVM, which shows to be a viable and more suitable option compared to two-class SVM to classify the data in this work. This is particularly valuable because of the nondeterministic nature of the negative interactions in GRN.

Across all classifiers, it is apparent that on average the use of RNA-seq data yielded higher recall values of more than 80% with all datasets and more than 90% with the majority of datasets. On the hypothesis that the RNA-seq normalization technique might affect the prediction accuracy, we noticed that TPM normalization shows a slightly overall improved performance than FPKM normalization. However, it cannot be stated that normalization techniques have any effect on accuracy.

**Figure 8:**
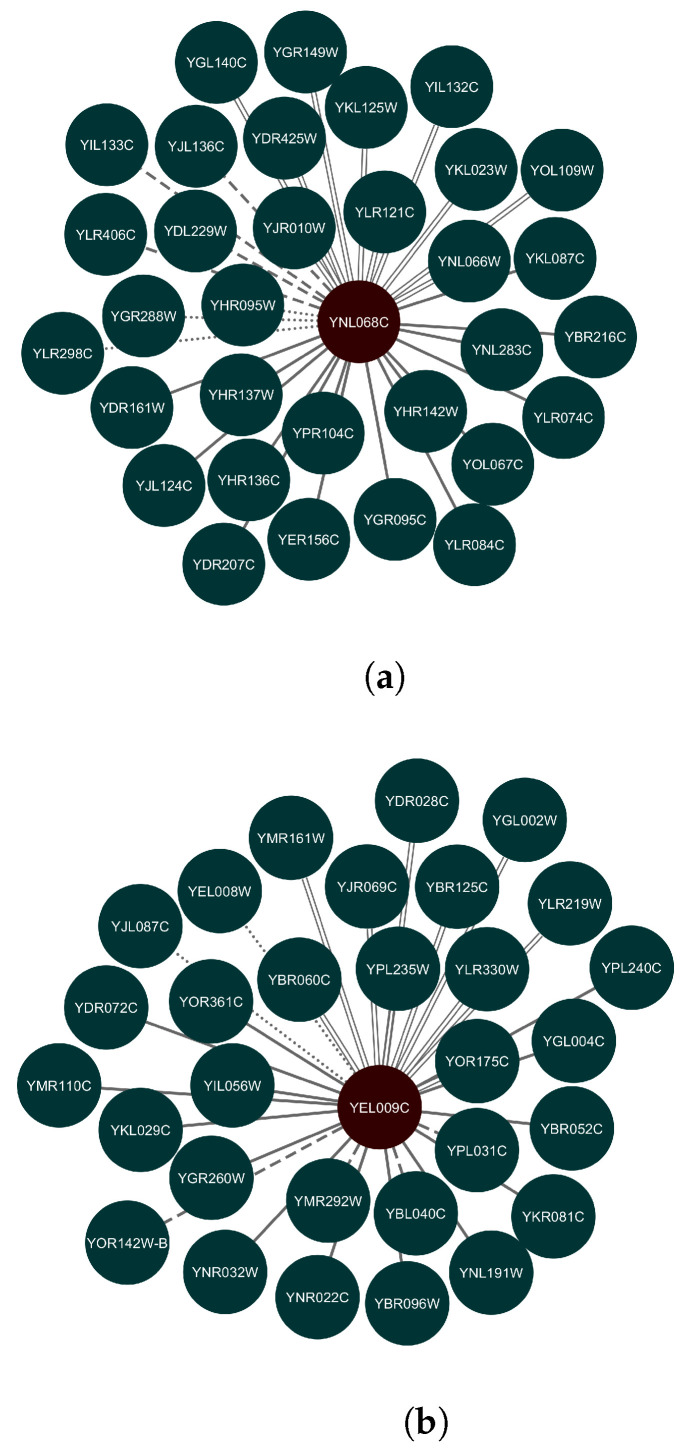
Two different transcription sub-networks show the TF gene and its target genes. (**a**) TF: **YNL068C**; (**b**) TF: **YEL009C**. The shared connections that are predicted by RNA-seq and microarray, and validated by Yeastract are represented in the graph by the *solid* lines. The *parallel* lines denote new potential target genes that are the shared predictions of RNA-seq and microarray but were not found in Yeastract. Unique RNA-seq and microarray predictions are represented by *dashed* and *dotted* lines, respectively.

Another appealing comparison measure is to compare the regulatory transcription networks constructed by Yeastract, RNA-seq using FPKM normalization, and microarray. We identified new potential targets for transcription factors using both profiling technologies, and we visualized the network modules using Cytoscape [33]. Figure 8a shows the network example for the transcription factor (TF) gene “YNL068C”. Another example network for the TF gene “YEL009C” is shown in Figure 8b. These networks represent part of a bigger transcription network showing the transcription factor genes and their target genes. Some of the network connections with the target genes are predicted by both RNA-seq and microarray and validated by Yeastract. We also show the shared predictions of microarray and RNA-seq that were not validated by Yeastract. Lastly, we represent the extra genes produced uniquely using RNA-seq and microarray data classification.

To evaluate our approach, we compared it to results reported previously (using related, but different datasets) for two other GRN inference methods (GRADIS [34] and GNE [35]). These methods have demonstrated superior performance over other well-known methods and also incorporate analysis of the yeast genome. Our evaluation metric was the area under the ROC curve, which was used as the primary performance metric in both GRN methods. The datasets used in both methods were sourced from the DREAM5 challenge, which includes 5091 genes [36]. In contrast, our approach utilized a more extensive dataset comprising 6041 genes, which includes all the genes listed in the DREAM5 challenge. The results presented in Table 2 demonstrate that our method outperforms the competing methods, indicating its superiority in predicting gene interactions.

## 4. Conclusions

In this article, we introduced a comparative approach to study and analyze the construction of a gene regulatory network (GRN) for *S. cerevisiae* based on profiling technologies: RNA-seq and microarray. The data for both technologies were acquired using samples with numerous experimental conditions. Each pair of genes was defined by a vector of 36 moments that represented the joint probability distribution of expression levels. Using various open-source GRN databases, gene categorization was predicted while employing different kernel classification models: one-class SVM, two-class SVM, and rare event classifier REWKLR. We reported the performance of both technologies across classifiers in terms of prediction accuracy, recall, precision, and other statistical measures. In general, RNA-seq outperformed microarray in several testing scenarios. We further addressed the effect of RNA-seq normalization techniques, such as TPM and FPKM on the overall performance. This work was an extension of our previous study [11] where we expanded it by combining and reporting the results using different datasets, benchmarks, and classification models. This method works well in capturing the gene interaction features from the gene expression data. We addressed the issue of determining negative gene interactions by using one-class SVM. We recommend the use of one-class classification in inferring GRN interactions to avoid the uncertainty of negative interactions.

Future work will expand the study of normalization techniques, including exploring RPKM. The study will also be extended to inferring the whole regulatory transcription network for multiple transcription factors and comparing the results to other studies. To improve accuracy, the integration of various data types, such as gene expression, epigenetic marks, chromatin accessibility, and transcription factor binding sites will be incorporated into the analysis. Furthermore, machine learning algorithms could enhance the accuracy of gene–disease association predictions by applying various feature selection techniques and integrating multiple data types. Causal inference methods may also be explored to identify causal relationships between genes and diseases, and network-based drug discovery approaches may be developed to use predicted disease-associated genes as potential drug targets. Furthermore, the potential use for studying microbiomes, possibly in a metagenomic/metatranscriptomic analysis is an interesting concept that can be developed in the future. These future directions could provide valuable insights into the molecular basis of disease and enable the development of more effective and personalized treatments.

## Figures and Tables

**Table 1:** The optimal training parameters and class accuracy for all classification models in the order (REWKLR, SVM, and one-class SVM).

**REWKLR**	***tpm***	***fpkm***	***micro***
Class Accuracy 0	94	94	97
Class Accuracy 1	83	95	75
λ	2.4	0.2	0.01
σ	0.2	0.1	1.5
**SVM**	***tpm***	***fpkm***	***micro***
Class Accuracy 0	99	99	93.5
Class Accuracy 1	86	85	99
γ	4	3.8	3.1
*C*	18	20	3.0
**One-class SVM**	***tpm***	***fpkm***	***micro***
Class Accuracy 1	96.6	97.5	94.8
γ	1.8	1.5	0.1
ν	0.03	0.03	0.2

**Table 2:** Comparative analysis based on the Area under the ROC Curve (AUC).

Methods	AUC
GNE [35]	0.825
GRADIS [34]	0.965
Our Method	0.981

## Data Availability

The data presented in this study are available in the Supplementary Materials.

## Supplementary Materials

The following supporting information can be downloaded at: https://www.mdpi.com/article/10.3390/biology12040518/s1, supplemental document includes descriptions of all other supplementary files and following figures, Figure S1: Microarray PR curve, Figure S2: RNA-seq TPM PR curve, Figure S3: RNA-seq FPKM PR curve, Figure S4: Bar Plot, Figure S5: Microarray ROC curve, Figure S6: RNA-seq TPM ROC curve, Figure S7: RNA-seq FPKM ROC curve.

## Abbreviations

The following abbreviations are used in this manuscript:

YEASTRACT	Yeast Search for Transcriptional Regulators and Consensus Tracking
YTRP	Yeast Transcriptional Regulatory Pathway
KEGG	Kyoto Encyclopedia of Genes and Genomes

## References

[1] Rai M., Tycksen E., Sandell L., Brophy R. (2018). Advantages of RNA-seq compared to RNA microarrays for transcriptome profiling of anterior cruciate ligament tears. J. Orthop. Res..

[2] Russo G., Zegar C., Giordano A. (2003). Advantages and limitations of microarray technology in human cancer. Oncogene.

[3] Koltai H., Weingarten-Baror C. (2008). Specificity of DNA microarray hybridization: Characterization, effectors and approaches for data correction. Nucleic Acids Res..

[4] Wang Z., Gerstein M., Snyder M. (2009). RNA-Seq: A revolutionary tool for transcriptomics. Nat. Rev. Genet..

[5] Ballouz S., Verleyen W., Gillis J. (2015). Guidance for RNA-seq co-expression network construction and analysis: Safety in numbers. Bioinformatics.

[6] Johnson K., Krishnan A. (2022). Robust normalization and transformation techniques for constructing gene coexpression networks from RNA-seq data. Genome Biol..

[7] Shahjaman M., Mollah M., Rahman M., Islam S., Mollah M. (2020). Robust identification of differentially expressed genes from RNA-seq data. Genomics.

[8] Zhang W., Yu Y., Hertwig F., Thierry-Mieg J., Zhang W., Thierry-Mieg D., Wang J., Furlanello C., Devanarayan V., Cheng J. (2015). Others Comparison of RNA-seq and microarray-based models for clinical endpoint prediction. Genome Biol..

[9] Giorgi F., Del Fabbro C., Licausi F. (2013). Comparative study of RNA-seq-and microarray-derived coexpression networks in *Arabidopsis thaliana*. Bioinformatics.

[10] Su Z., Fang H., Hong H., Shi L., Zhang W., Zhang W., Zhang Y., Dong Z., Lancashire L., Bessarabova M. (2014). An investigation of biomarkers derived from legacy microarray data for their utility in the RNA-seq era. Genome Biol..

[11] Al-Aamri A., Taha K., Maalouf M., Kudlicki A., Homouz D. (2020). Inferring Causation in Yeast gene association Networks with Kernel Logistic Regression. Evol. Bioinform..

[12] Edgar R., Domrachev M., Lash A. (2002). Gene Expression Omnibus: *NCBI* gene expression and hybridization array data repository. Nucleic Acids Res..

[13] Shumway M., Cochrane G., Sugawara H. (2010). Archiving next generation sequencing data. Nucleic Acids Res..

[14] SRA Toolkit Development Team Sequence Read Archive Toolkit. https://trace.ncbi.nlm.nih.gov/Traces/sra/sra.cgi?view=software.

[15] Li B., Dewey C. (2011). RSEM: Accurate transcript quantification from RNA-Seq data with or without a reference genome. BMC Bioinform..

[16] Langmead B., Trapnell C., Pop M., Salzberg S. (2009). Ultrafast and memory-efficient alignment of short DNA sequences to the human genome. Genome Biol..

[17] Cunningham F., Allen J., Allen J., Alvarez-Jarreta J., Amode M., Armean I., Austine-Orimoloye O., Azov A., Barnes I., Bennett R. (2022). Ensembl 2022. Nucleic Acids Res..

[18] Consortium U. (2021). UniProt: The universal protein knowledgebase in 2021. Nucleic Acids Res..

[19] Jackson J. (2005). A User’s Guide to Principal Components.

[20] Teixeira M., Monteiro P., Jain P., Tenreiro S., Fernandes A., Mira N., Alenquer M., Freitas A., Oliveira A., Sá-Correia I. (2006). The YEASTRACT database: A tool for the analysis of transcription regulatory associations in *Saccharomyces cerevisiae*. Nucleic Acids Res..

[21] Yang T., Wang C., Wang Y., Wu W. (2014). YTRP: A repository for yeast transcriptional regulatory pathways. Database.

[22] Harbison C., Gordon D., Lee T., Rinaldi N., Macisaac K., Danford T., Hannett N., Tagne J., Reynolds D., Yoo J. (2004). Transcriptional regulatory code of a eukaryotic genome. Nature.

[23] Cherry J., Hong E., Amundsen C., Balakrishnan R., Binkley G., Chan E., Christie K., Costanzo M., Dwight S., Engel S. (2012). Saccharomyces Genome Database: The genomics resource of budding yeast. Nucleic Acids Res..

[24] Kanehisa M., Furumichi M., Sato Y., Kawashima M., Ishiguro-Watanabe M. (2022). KEGG for taxonomy-based analysis of pathways and genomes. Nucleic Acids Res..

[25] Maalouf M., Humouz D., Kudlicki A. (2014). Robust weighted kernel logistic regression to predict gene-gene regulatory association. IIE Annu. Conf. Proc..

[26] Maalouf M., Trafalis T. (2011). Robust weighted kernel logistic regression in imbalanced and rare events data. Comput. Stat. Data Anal..

[27] Maalouf M., Homouz D. (2014). Kernel ridge regression using truncated newton method. Knowl.-Based Syst..

[28] Köknar-Tezel S., Latecki L. (2009). Improving SVM classification on imbalanced data sets in distance spaces. IEEE Int. Conf. Data Min..

[29] Azeem M., Jamil M., Shang Y. (2023). Notes on the localization of generalized hexagonal cellular networks. Mathematics.

[30] Schölkopf B., Williamson R., Smola A., Shawe-Taylor J., Platt J. (1999). Support vector method for novelty detection. Adv. Neural Inf. Process. Syst..

[31] Guerbai Y., Chibani Y., Hadjadji B. (2014). The effective use of the One-Class SVM classifier for reduced training samples and its application to handwritten signature verification. Int. Conf. Multimed. Comput. Syst..

[32] Pedregosa F., Varoquaux G., Gramfort A., Michel V., Thirion B., Grisel O., Blondel M., Prettenhofer P., Weiss R., Dubourg V. (2011). Scikit-learn: Machine learning in Python. J. Mach. Learn. Res..

[33] Shannon P., Markiel A., Ozier O., Baliga N., Wang J., Ramage D., Amin N., Schwikowski B., Ideker T. (2003). Cytoscape: A software environment for integrated models of biomolecular interaction networks. Genome Res..

[34] Razaghi-Moghadam Z., Nikoloski Z. (2020). Supervised learning of gene-regulatory networks based on graph distance profiles of transcriptomics data. NPJ Syst. Biol. Appl..

[35] Kc K., Li R., Cui F., Yu Q., Haake A. (2019). GNE: A deep learning framework for gene network inference by aggregating biological information. BMC Syst. Biol..

[36] Marbach D., Costello J.C., Küffner R., Vega N.M., Prill R.J., Camacho D.M., Allison K.R., Kellis M., Collins J.J., Stolovitzky G. (2012). Wisdom of crowds for robust gene network inference. Nat. Methods.

