# Supplementary material for: Inferring Gene Regulatory Networks from RNA-seq Data Using Kernel Classification
Source: Biology (Basel). 2023 Mar 29;12(4):518. doi: 10.3390/biology12040518 (PMC10135911; doi:10.3390/biology12040518)
Supplement: Supplementary file 1 [file biology-12-00518-s001.zip › biology-2211468-supplementary/Supplementary/Supplemental Document.pdf]

Inferring Gene Regulatory Networks from RNA-seq Data using Kernel Classification: supplemental document

This supplementary materials document contains additional figures, files, and background. The sections are as follows: Descriptions of other supplementary files, additional figures, and an in-depth comparison between RNA-seq and microarray.

A. Descriptions of other supplementary files

The description for each file included is mentioned in the following:

- **"SRA List.xlsx":** The SRA accession numbers used to download RNA-seq count reads data via RSEM
- **"Micro-training-pca-36.csv":** The data matrix of 10k zeros followed by 10k ones represented by 36 moments for micorarray.
- **"TPM-training-pca-36.csv":** The data matrix of 10k zeros followed by 10k ones represented by 36 moments for RNA-seq (TPM normalized).
- **"FPKM-training-pca-36.csv":** The data matrix of 10k zeros followed by 10k ones represented by 36 moments for RNA-seq (FPKM normalized).
- **"Detailed Data of Networks.xlsx":** These are the genes predicted for the networks of TF *YEL009C* and TF *YNL068C*. Part of the network is presented in Figure 8 of the main text. The first column lists the shared predictions between RNA-seq and microarray. The second column lists unique predictions by RNA-seq. The last column lists unique predictions by microarray.

B. Figures

The following figures show the precision-recall curve with shaded error bars for each dataset using REWKLR and SVM. Figure S1 illustrates the microarray dataset performance.

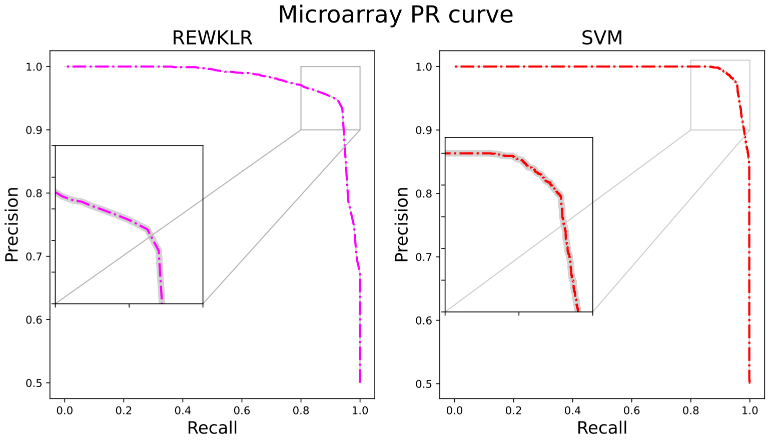

Figure S1. 2-class Precision-Recall curves for REWKLR and SVM.

Figure S2 illustrates the RNA-seq TPM normalization dataset.

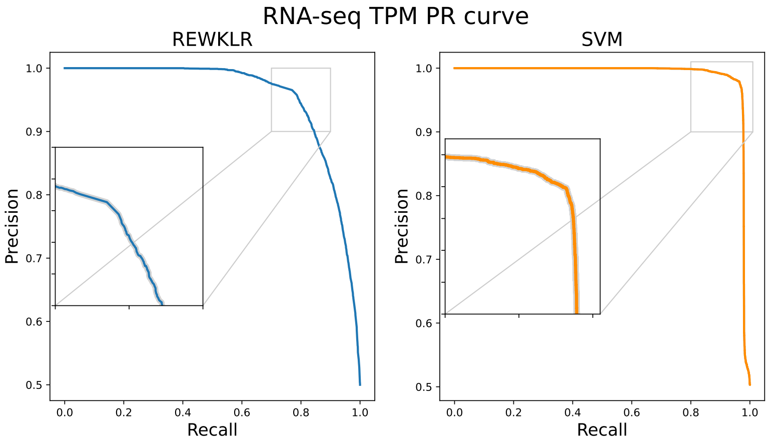

Figure S2. 2-class Precision-Recall curves for REWKLR and SVM.

Figure S3 illustrates the RNA-seq FPKM normalization dataset.

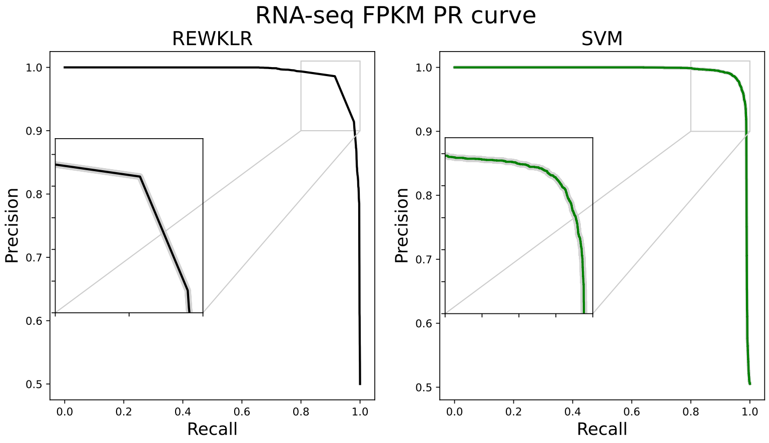

Figure S3. 2-class Precision-Recall curves for REWKLR and SVM.

The bar plot in Figure S4 demonstrates the performance of each classifier using all testing datasets with a zoomed version for a better view of the error bars.

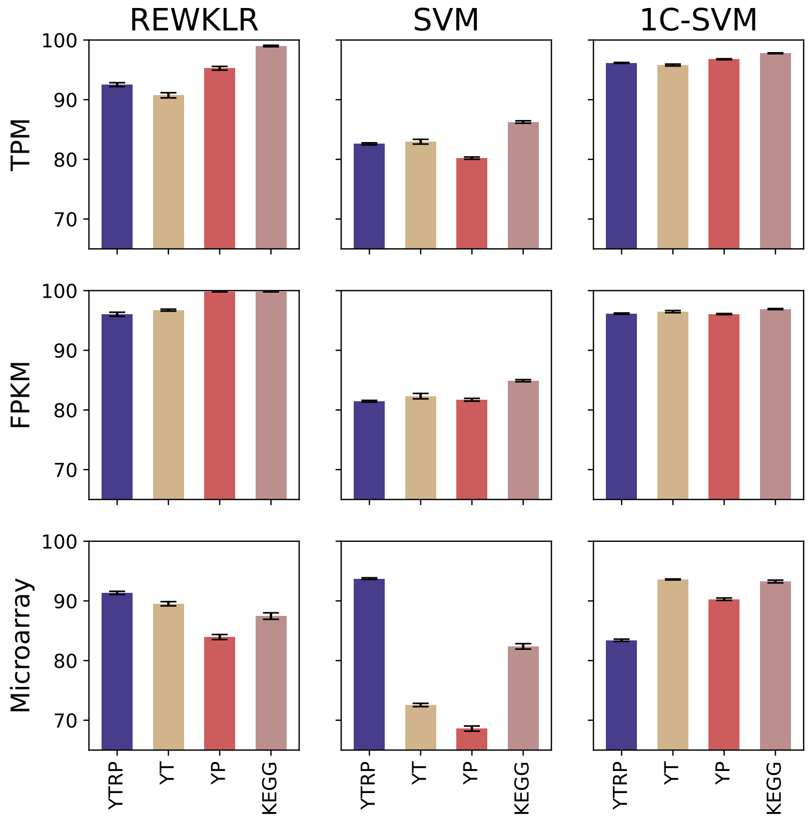

Figure S4. The performance results is reported for the combination of all datasets, benchmarks, and classification models in terms of average recall. That resulted in curating twelve different testing datasets used for prediction. Each dataset (RNA-seq TPM normalized, RNA-seq FPKM normalized, and microarray) was validated using four different benchmarks (YTRP[21], YT[20], YP[23], and KEGG[24]). The curated datasets were classified and evaluated using classifiers (REWKLR, SVM, and one-class SVM). The error bars are considerably small.

The following figures show the Receiver Operating Characteristic (ROC) curves with shaded error bars for each dataset using REWKLR and SVM. Figure S5 shows the microarray dataset.

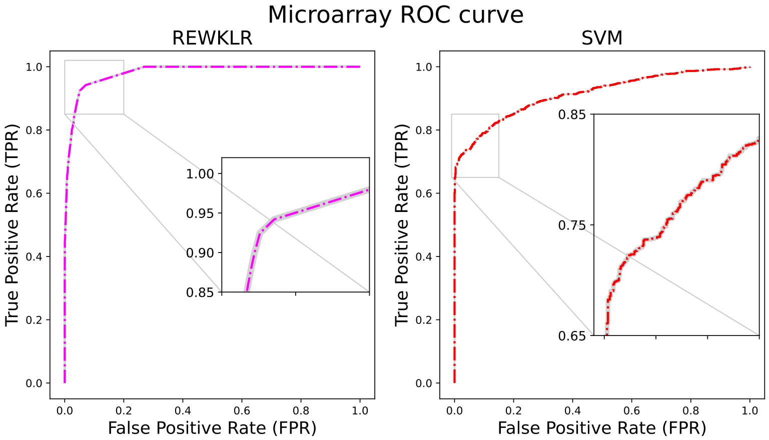

Figure S5. 2-class ROC curves for REWKLR and SVM.

Figure S6 illustrates the RNA-seq TPM normalization dataset.

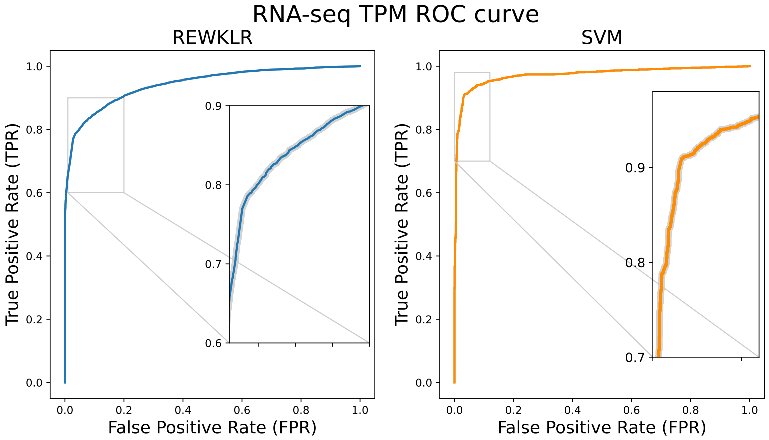

Figure S6. 2-class ROC curves for REWKLR and SVM.

Figure S7 illustrates the RNA-seq FPKM normalization dataset.

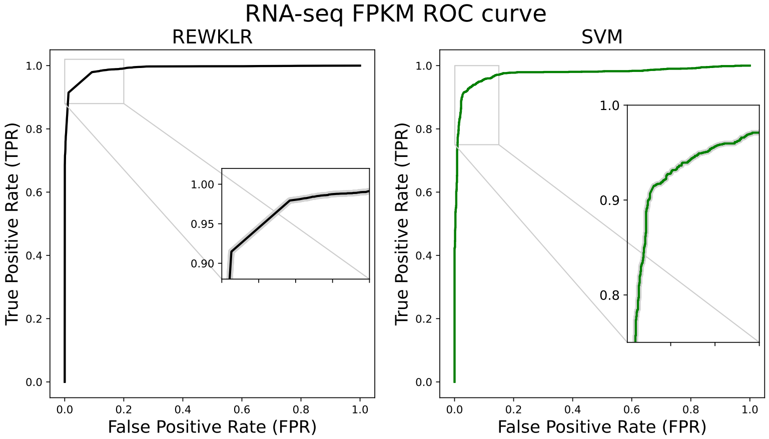

Figure S7. 2-class ROC curves for REWKLR and SVM.

C. Comparison: RNA-seq vs microarray

The advantages of RNA-seq and microarray have been demonstrated by various concordance and comparative analysis studies. The potential of RNA-seq in identifying low-abundant transcripts and isoforms with biological significance has been shown through a concordance study evaluating the gene expression profile on time-from-injury of anterior cruciate ligament tissues and a rat liver differential gene expression study evaluating the effect of different chemicals [1, 37]. Comparative studies using The Cancer Genome Atlas (TCGA) observed that RNA-seq includes comprehensive details on particular transcript expression patterns and noticed some discrepancies for the transcripts with extremely high or low expression levels in microarray analysis [8, 38]. Although most of the comparative studies between microarray and RNA-seq data showed an overall agreement on DEGs identified through both technologies, the validation studies using RT-PCR observed a higher coincidence rate for the RNA-seq generated data. [39, 40].

Studies have also reported RNA-seq outperforms microarray in some analyses such as isoform characterization, toxicogenomics evaluation, cellular dynamics upon altered gravity exposure, and genome-level alternative splicing [41–43]. A toxicogenomics study using rat liver RNA demonstrated a 78% overlap of DEGs identified between microarray and RNA-seq. The study observed that RNA-seq provides more insight into mechanisms of toxicity due to the advantages such as more identified DEGs which suggested modulation of additional liver relevant pathways, non-coding DEGs, and wider expression level ranges which aided in improved mechanistic understanding [41]. Even though both technologies provide information on splice events, advantages of RNA-seq like improved sensitivity in finding genes with extremely low expressions and better approximation of gene/transcript concentrations suggest it is a better technology to identify the splice variants [42]. Similarly, a transcriptome profiling study on Jurkat T cell RNA samples to understand the cellular dynamics upon altered gravity exposure demonstrated overall comparability between both data but RNA-seq showed a greater sensitivity [40]. Based on the studies reported, RNA-seq-based transcriptome profiling overcomes the limitations of microarrays such as cross and non-specific hybridization, and limited probe-level detection which also makes it the technology of choice by the scientific community [37, 42, 44].

REFERENCES

Citations that are relevant to the primary manuscript and the supplementary document are included in both places.

REFERENCES

1. Rai, M.; Tycksen, E.; Sandell, L.; Brophy, R. Advantages of RNA-seq compared to RNA microarrays for transcriptome profiling of anterior cruciate ligament tears. *J. Orthop. Res.* **2018**, *36*, 484–497.

2. Russo, G.; Zegar, C.; Giordano, A. Advantages and limitations of microarray technology in human cancer. *Oncogene* **2003**, *22*, 6497–6507.
3. Koltai, H.; Weingarten-Baror, C. Specificity of DNA microarray hybridization: Characterization, effectors and approaches for data correction. *Nucleic Acids Res.* **2008**, *36*, 2395–2405.
4. Wang, Z.; Gerstein, M.; Snyder, M. RNA-Seq: A revolutionary tool for transcriptomics. *Nat. Rev. Genet.* **2009**, *10*, 57–63.
5. Ballouz, S.; Verleyen, W.; Gillis, J. Guidance for RNA-seq co-expression network construction and analysis: Safety in numbers. *Bioinformatics* **2015**, *31*, 2123–2130.
6. Johnson, K.; Krishnan, A. Robust normalization and transformation techniques for constructing gene coexpression networks from RNA-seq data. *Genome Biol.* **2022**, *23*, 1–26.
7. Shahjaman, M.; Mollah, M.; Rahman, M.; Islam, S.; Mollah, M. Robust identification of differentially expressed genes from RNA-seq data. *Genomics* **2020**, *112*, 2000–2010.
8. Zhang, W.; Yu, Y.; Hertwig, F.; Thierry-Mieg, J.; Zhang, W.; Thierry-Mieg, D.; Wang, J.; Furlanello, C.; Devanarayan, V.; Cheng, J. Others Comparison of RNA-seq and microarray-based models for clinical endpoint prediction. *Genome Biol.* **2015**, *16*, 1–12.
9. Giorgi, F.; Del Fabbro, C.; Licausi, F. Comparative study of RNA-seq-and microarray-derived coexpression networks in *Arabidopsis thaliana*. *Bioinformatics* **2013**, *29*, 717–724.
10. Su, Z.; Fang, H.; Hong, H.; Shi, L.; Zhang, W.; Zhang, W.; Zhang, Y.; Dong, Z.; Lancashire, L.; Bessarabova, M.; et al. An investigation of biomarkers derived from legacy microarray data for their utility in the RNA-seq era. *Genome Biol.* **2014**, *15*, 1–25.
11. Al-Aamri, A.; Taha, K.; Maalouf, M.; Kudlicki, A.; Homouz, D. Inferring Causation in Yeast gene association Networks with Kernel Logistic Regression. *Evol. Bioinform.* **2020**, *16*, 1–6.
12. Edgar, R.; Domrachev, M.; Lash, A. Gene Expression Omnibus: *NCBI* gene expression and hybridization array data repository. *Nucleic Acids Res.* **2002**, *30*, 207–210.
13. Shumway, M.; Cochrane, G.; Sugawara, H. Archiving next generation sequencing data. *Nucleic Acids Res.* **2010**, *38*, D870–D871.
14. SRA Toolkit Development Team Sequence Read Archive Toolkit. Available online: https://trace.ncbi.nlm.nih.gov/Traces/sra/sra.cgi?view=software (accessed on 10 August 2022).
15. Li, B.; Dewey, C. RSEM: Accurate transcript quantification from RNA-Seq data with or without a reference genome. *BMC Bioinform.* **2011**, *12*, 1–16.
16. Langmead, B.; Trapnell, C.; Pop, M.; Salzberg, S. Ultrafast and memory-efficient alignment of short DNA sequences to the human genome. *Genome Biol.* **2009**, *10*, 1–10.
17. Cunningham, F.; Allen, J.; Allen, J.; Alvarez-Jarreta, J.; Amode, M.; Armean, I.; Austine-Orimoloye, O.; Azov, A.; Barnes, I.; Bennett, R.; et al. Ensembl 2022. *Nucleic Acids Res.* **2022**, *50*, D988–D995.
18. Consortium, U. UniProt: The universal protein knowledgebase in 2021. *Nucleic Acids Res.* **2021**, *49*, D480–D489.
19. Jackson, J. *A User's Guide to Principal Components*; John Wiley & Sons: Hoboken, NJ, USA, **2005**.
20. Teixeira, M.; Monteiro, P.; Jain, P.; Tenreiro, S.; Fernandes, A.; Mira, N.; Alenquer, M.; Freitas, A.; Oliveira, A.; Sá-Correia, I. The YEASTRACT database: A tool for the analysis of transcription regulatory associations in *Saccharomyces cerevisiae*. *Nucleic Acids Res.* **2006**, *34*, D446–D451.
21. Yang, T.; Wang, C.; Wang, Y.; Wu, W. YTRP: A repository for yeast transcriptional regulatory pathways. *Database* **2014**, *2014*, bau014 .
22. Harbison, C.; Gordon, D.; Lee, T.; Rinaldi, N.; Macisaac, K.; Danford, T.; Hannett, N.; Tagne, J.; Reynolds, D.; Yoo, J.; et al. Transcriptional regulatory code of a eukaryotic genome. *Nature* **2004**, *431*, 99–104.
23. Cherry, J.; Hong, E.; Amundsen, C.; Balakrishnan, R.; Binkley, G.; Chan, E.; Christie, K.; Costanzo, M.; Dwight, S.; Engel, S.; et al. Saccharomyces Genome Database: The genomics resource of budding yeast. *Nucleic Acids Res.* **2012**, *40*, D700–D705.
24. Kanehisa, M.; Furumichi, M.; Sato, Y.; Kawashima, M.; Ishiguro-Watanabe, M. KEGG for taxonomy-based analysis of pathways and genomes. *Nucleic Acids Res.* **2022**, *51*, D587–D592.
25. Maalouf, M.; Humouz, D.; Kudlicki, A. Robust weighted kernel logistic regression to predict gene-gene regulatory association. *IIE Annu. Conf. Proc.* **2014**, *2014*, 1356–1360.
26. Maalouf, M.; Trafalis, T. Robust weighted kernel logistic regression in imbalanced and rare events data. *Comput. Stat. Data Anal.* **2011**, *55*, 168–183.
27. Maalouf, M.; Homouz, D. Kernel ridge regression using truncated newton method. *Knowl.-Based Syst.* **2014**, *71* 339–344.

28. Köknar-Tezel, S.; Latecki, L. Improving SVM classification on imbalanced data sets in distance spaces. *IEEE Int. Conf. Data Min.* **2009**, *2009*, 259–267.
29. Azeem, M.; Jamil, M.; Shang, Y. Notes on the localization of generalized hexagonal cellular networks. *Mathematics* **2023**, *11*, 844.
30. Schölkopf, B.; Williamson, R.; Smola, A.; Shawe-Taylor, J.; Platt, J. Support vector method for novelty detection. *Adv. Neural Inf. Process. Syst.* **1999**, *12*, 1–7.
31. Guerbai, Y.; Chibani, Y.; Hadjadji, B. The effective use of the One-Class SVM classifier for reduced training samples and its application to handwritten signature verification. *Int. Conf. Multimed. Comput. Syst.* **2014**, *2014*, 362–366.
32. Pedregosa, F.; Varoquaux, G.; Gramfort, A.; Michel, V.; Thirion, B.; Grisel, O.; Blondel, M.; Prettenhofer, P.; Weiss, R.; Dubourg, V.; et al. Scikit-learn: Machine learning in Python. *J. Mach. Learn. Res.* **2011**, *12*, 2825–2830.
33. Shannon, P.; Markiel, A.; Ozier, O.; Baliga, N.; Wang, J.; Ramage, D.; Amin, N.; Schwikowski, B.; Ideker, T. Cytoscape: A software environment for integrated models of biomolecular interaction networks. *Genome Res.* **2003**, *13*, 2498–2504.
34. Razaghi-Moghadam, Z.; Nikoloski, Z. Supervised learning of gene-regulatory networks based on graph distance profiles of transcriptomics data. *NPJ Syst. Biol. Appl.* **2020**, *6*, 21.
35. Kc, K.; Li, R.; Cui, F.; Yu, Q.; Haake, A. GNE: A deep learning framework for gene network inference by aggregating biological information. *BMC Syst. Biol.* **2019**, *13*, 1–14.
36. Marbach, D.; Costello, J.C.; Küffner, R.; Vega, N.M.; Prill, R.J.; Camacho, D.M.; Allison, K.R.; Kellis, M.; Collins, J.J.; Stolovitzky, G. Wisdom of crowds for robust gene network inference. *Nat. Methods* **2012**, *9*, 796–804.
37. Wang, C., Gong, B., Bushel, P., Thierry-Mieg, J., Thierry-Mieg, D., Xu, J., Fang, H., Hong, H., Shen, J., Su, Z. & Others The concordance between RNA-seq and microarray data depends on chemical treatment and transcript abundance. *Nature Biotechnology*. **32**, 926-932 (2014)
38. Chen, L., Sun, F., Yang, X., Jin, Y., Shi, M., Wang, L., Shi, Y., Zhan, C. & Wang, Q. Correlation between RNA-Seq and microarrays results using TCGA data. *Gene*. **628** pp. 200-204 (2017)
39. Li, J., Hou, R., Niu, X., Liu, R., Wang, Q., Wang, C., Li, X., Hao, Z., Yin, G. & Zhang, K. Comparison of microarray and RNA-Seq analysis of mRNA expression in dermal mesenchymal stem cells. *Biotechnology Letters*. **38**, 33-41 (2016)
40. Vahlensieck, C., Thiel, C., Adelmann, J., Lauber, B., Polzer, J. & Ullrich, O. Rapid transient transcriptional adaptation to hypergravity in jurkat T cells revealed by comparative analysis of microarray and RNA-seq data. *International Journal Of Molecular Sciences*. **22**, 8451 (2021)
41. Rao, M., Van Vleet, T., Ciurlionis, R., Buck, W., Mittelstadt, S., Blomme, E. & Liguori, M. Comparison of RNA-Seq and microarray gene expression platforms for the toxicogenomic evaluation of liver from short-term rat toxicity studies. *Frontiers In Genetics*. **9** pp. 636 (2019)
42. Romero, J., Ortiz-Estévez, M., Muniategui, A., Carrancio, S., Miguel, F., Carazo, F., Montuenga, L., Loos, R., Pio, R., Trotter, M. & Others Comparison of RNA-seq and microarray platforms for splice event detection using a cross-platform algorithm. *BMC Genomics*. **19**, 1-14 (2018)
43. Zhao, S., Fung-Leung, W., Bittner, A., Ngo, K. & Liu, X. Comparison of RNA-Seq and microarray in transcriptome profiling of activated T cells. *PloS One*. **9**, e78644 (2014)
44. Fumagalli, D., Blanchet-Cohen, A., Brown, D., Desmedt, C., Gacquer, D., Michiels, S., Rothé, F., Majjaj, S., Salgado, R., Larsimont, D. & Others Transfer of clinically relevant gene expression signatures in breast cancer: from Affymetrix microarray to Illumina RNA-Sequencing technology. *BMC Genomics*. **15**, 1-12 (2014)
