# Supplementary figures and images for: Inferring Gene Regulatory Networks from RNA-seq Data Using Kernel Classification
Source: Biology (Basel). 2023 Mar 29;12(4):518. doi: 10.3390/biology12040518 (PMC10135911; doi:10.3390/biology12040518)

# Microarray PR curve

## REWKLR

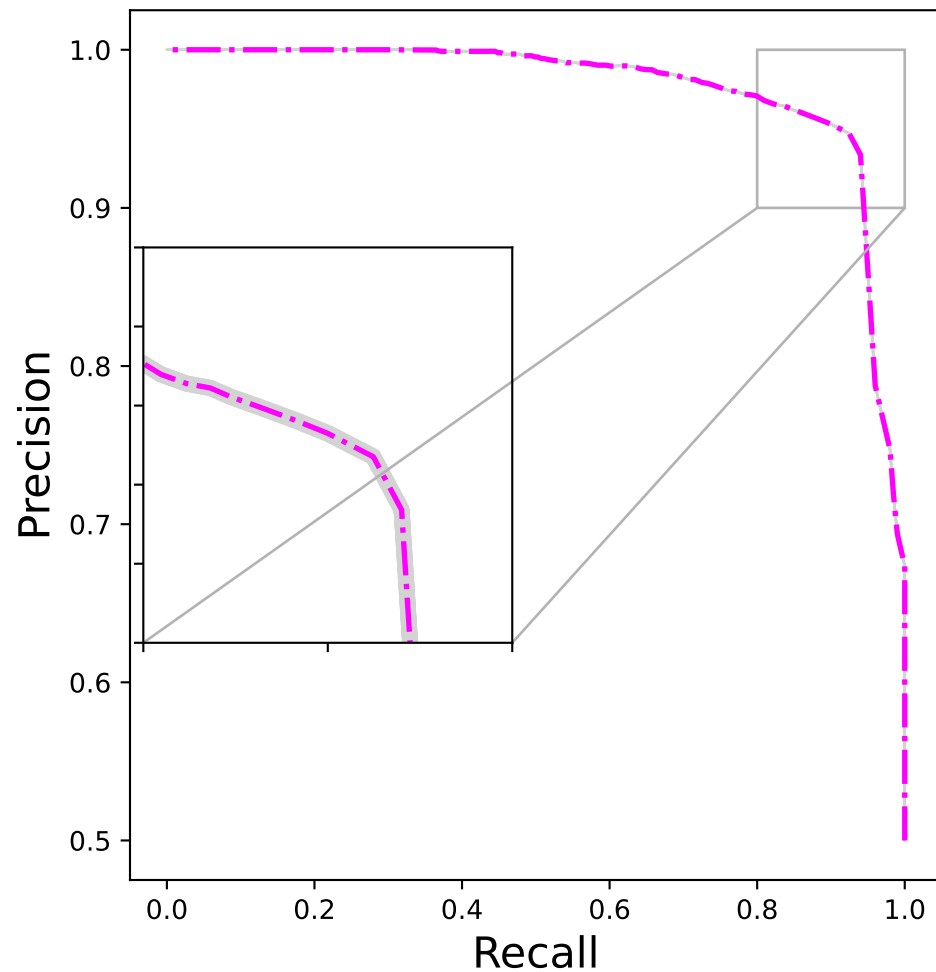

## SVM

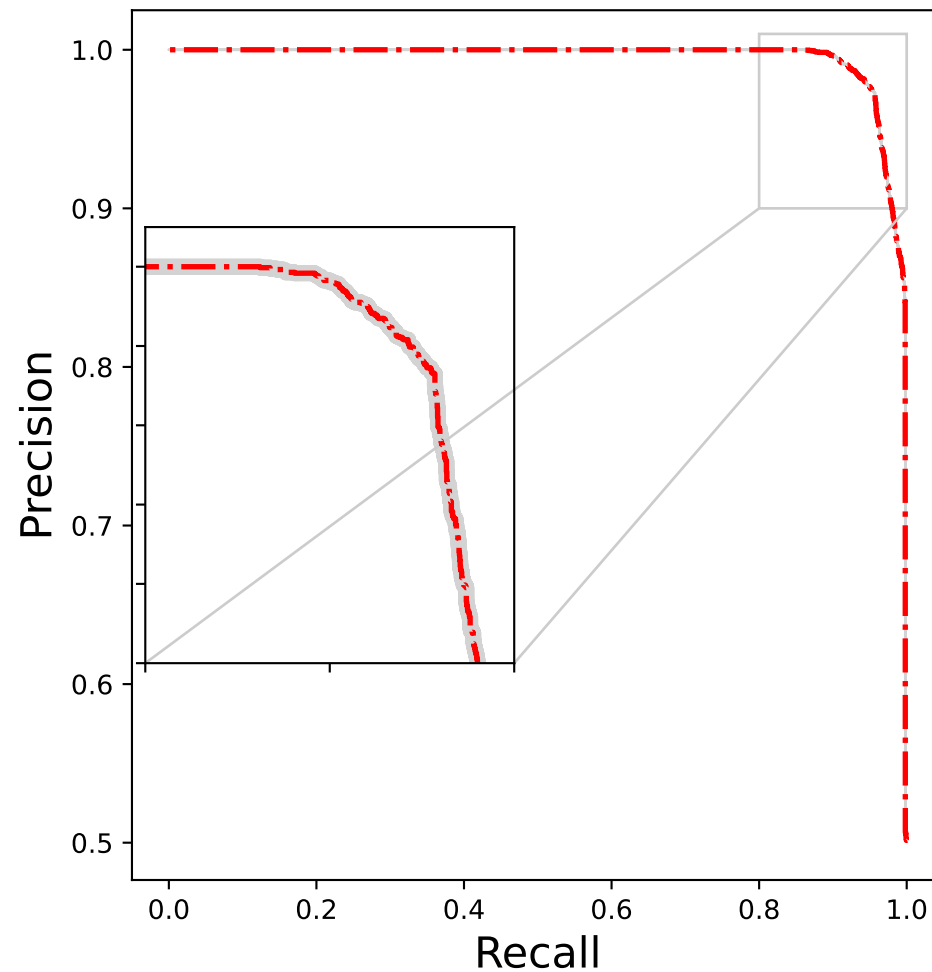

Supplement: Supplementary file 1 [file biology-12-00518-s001.zip › biology-2211468-supplementary/Supplementary/Figure S1.pdf]

# RNA-seq TPM PR curve

## REWKLR

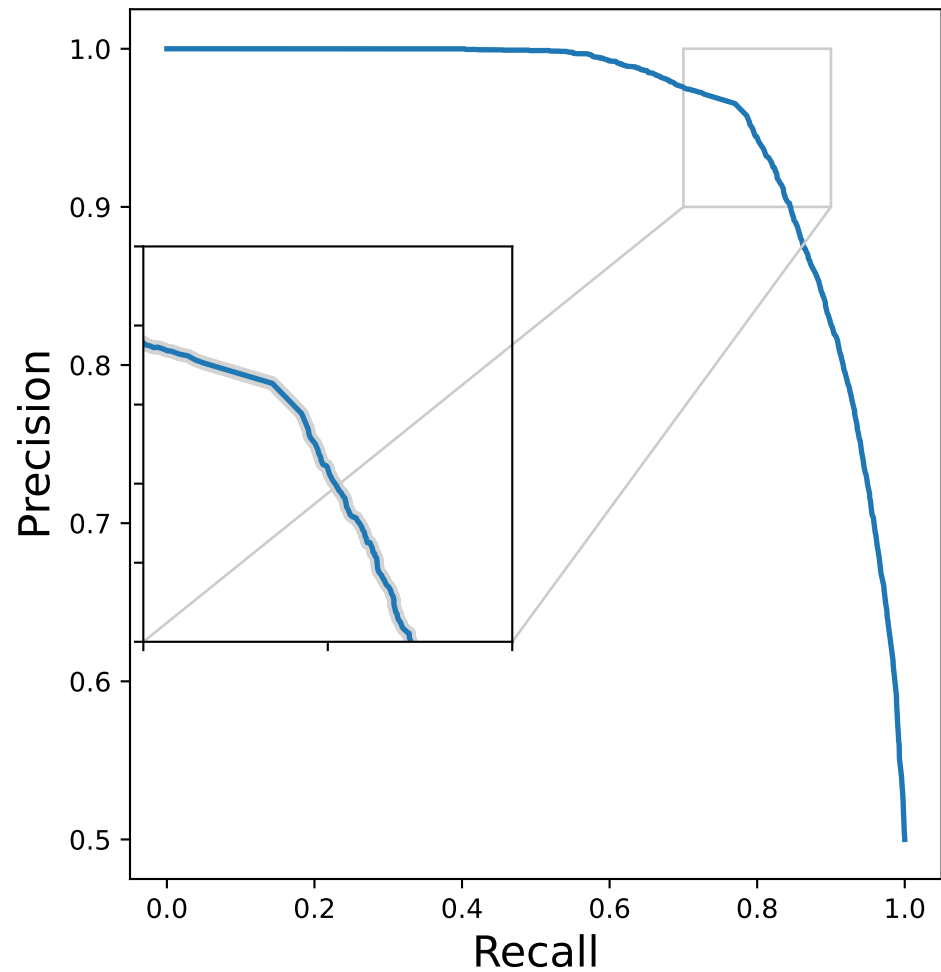

## SVM

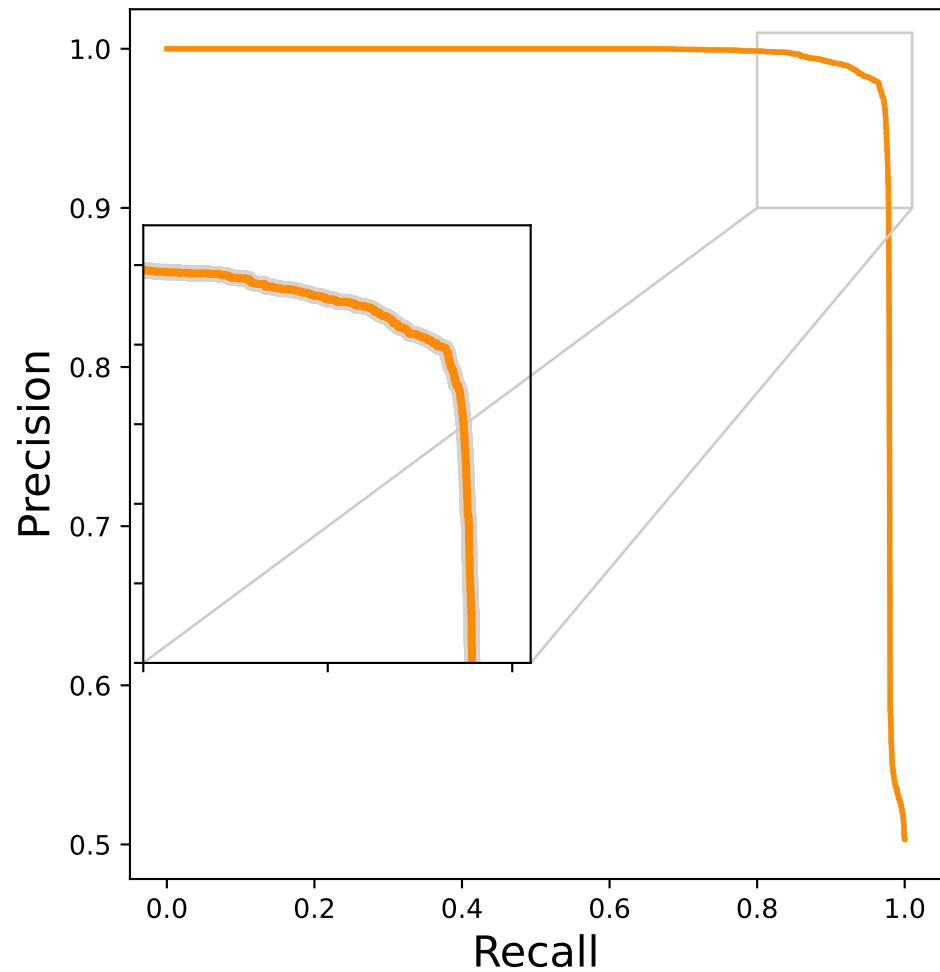

Supplement: Supplementary file 1 [file biology-12-00518-s001.zip › biology-2211468-supplementary/Supplementary/Figure S2.pdf]

# RNA-seq FPKM PR curve

## REWKLR

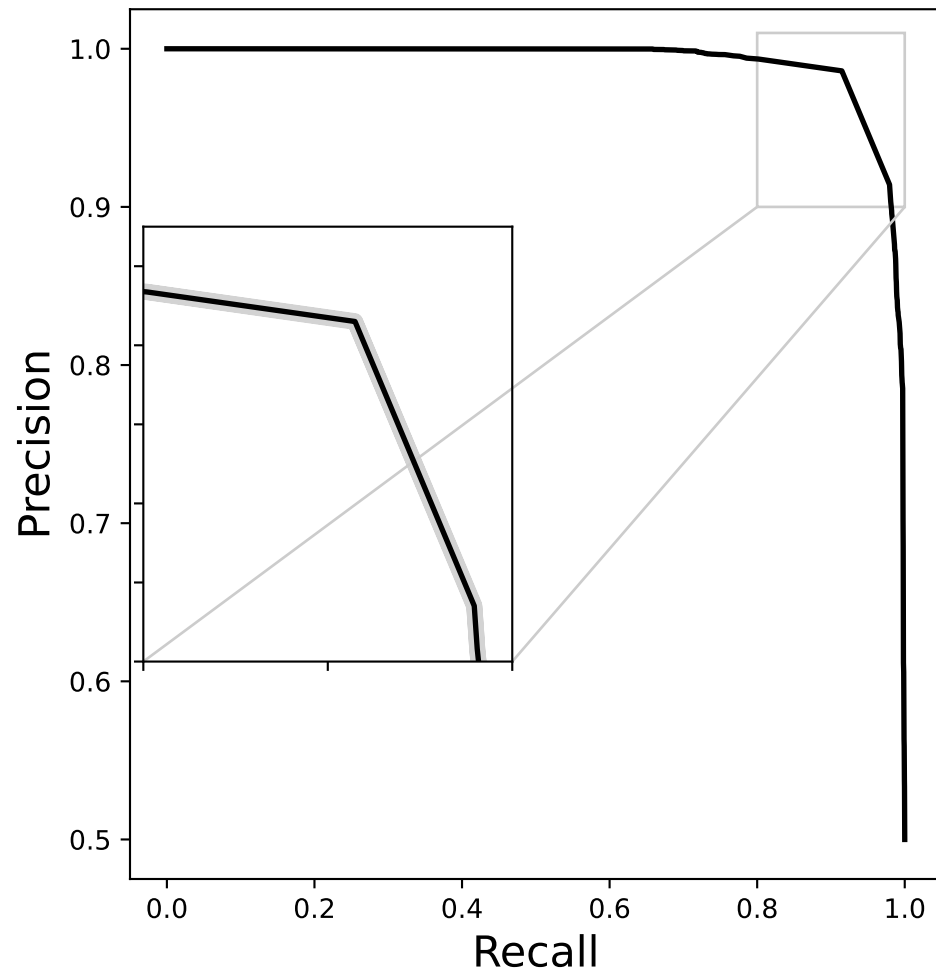

## SVM

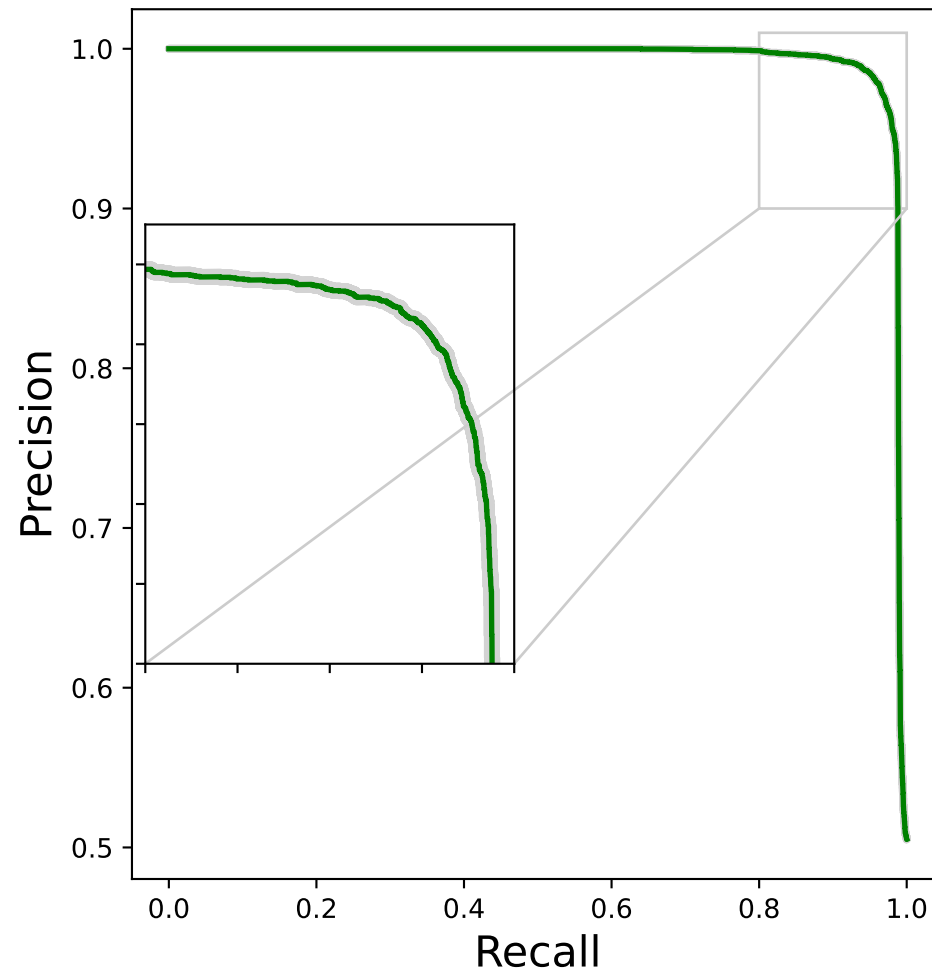

Supplement: Supplementary file 1 [file biology-12-00518-s001.zip › biology-2211468-supplementary/Supplementary/Figure S3.pdf]

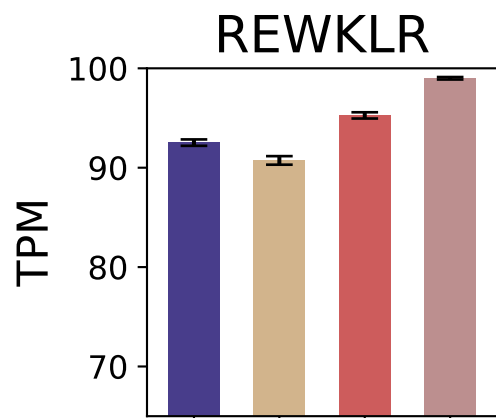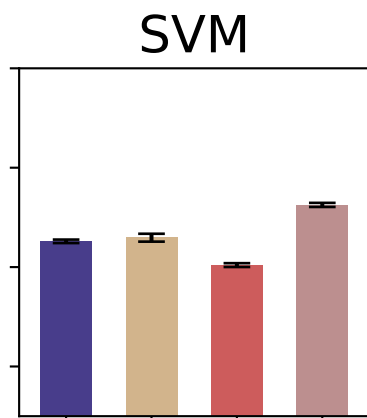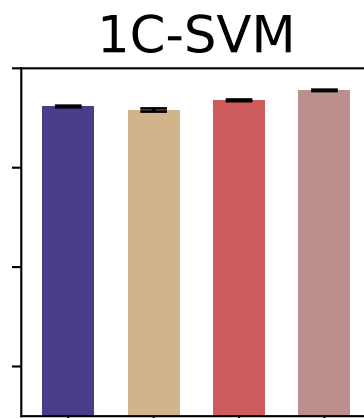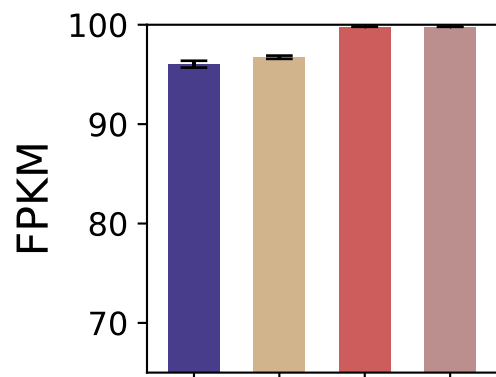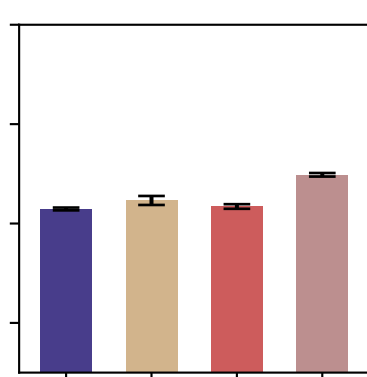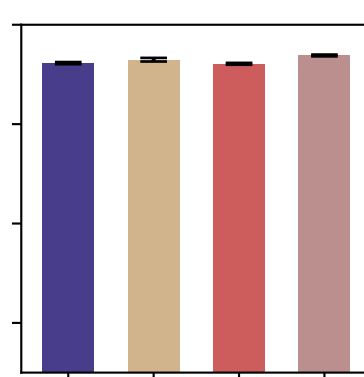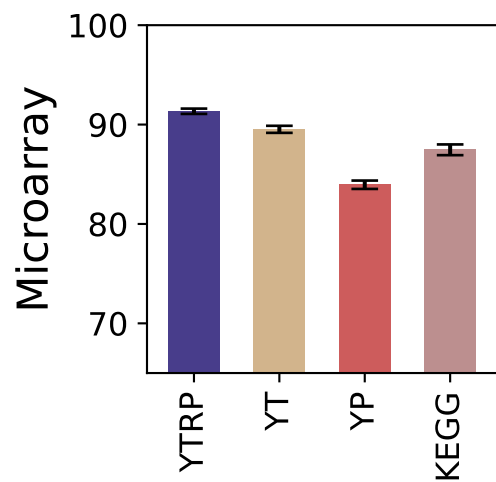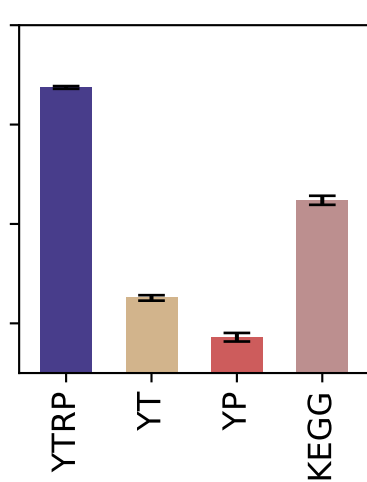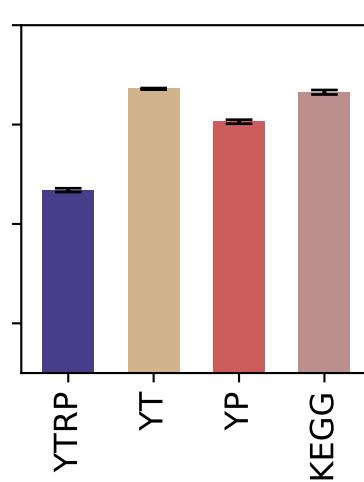

Supplement: Supplementary file 1 [file biology-12-00518-s001.zip › biology-2211468-supplementary/Supplementary/Figure S4.pdf]

# Microarray ROC curve

## REWKLR

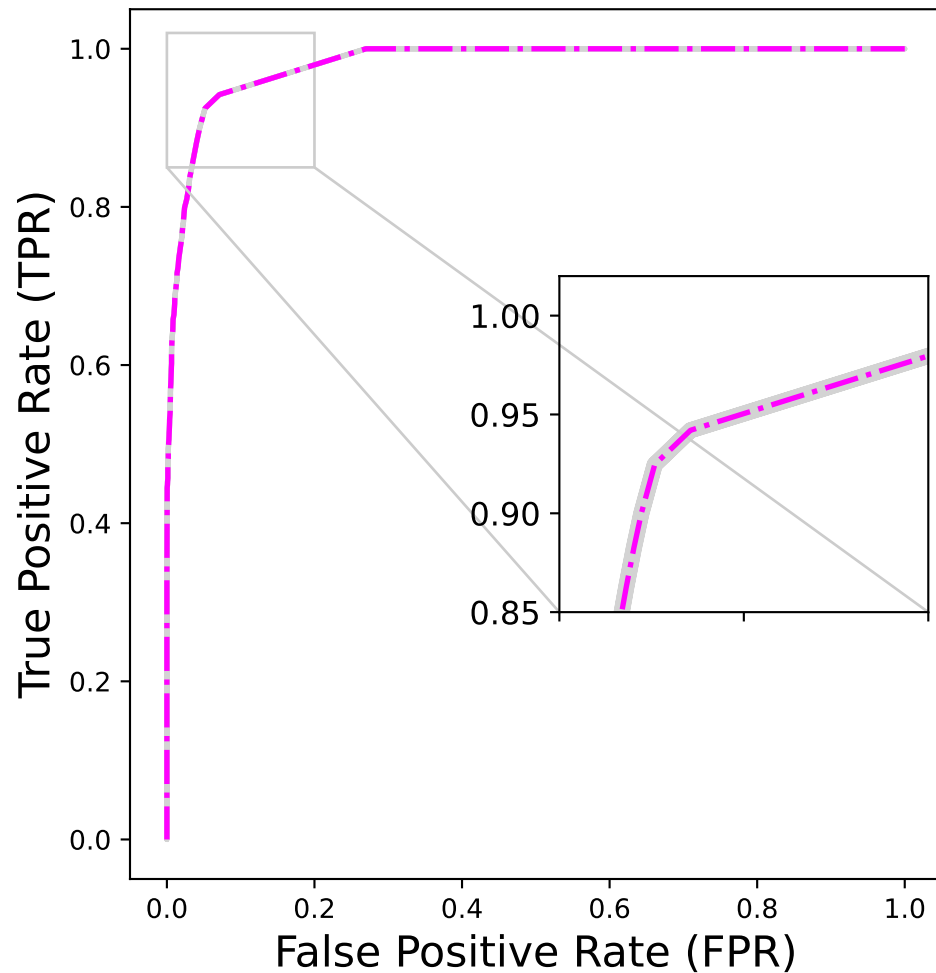

## SVM

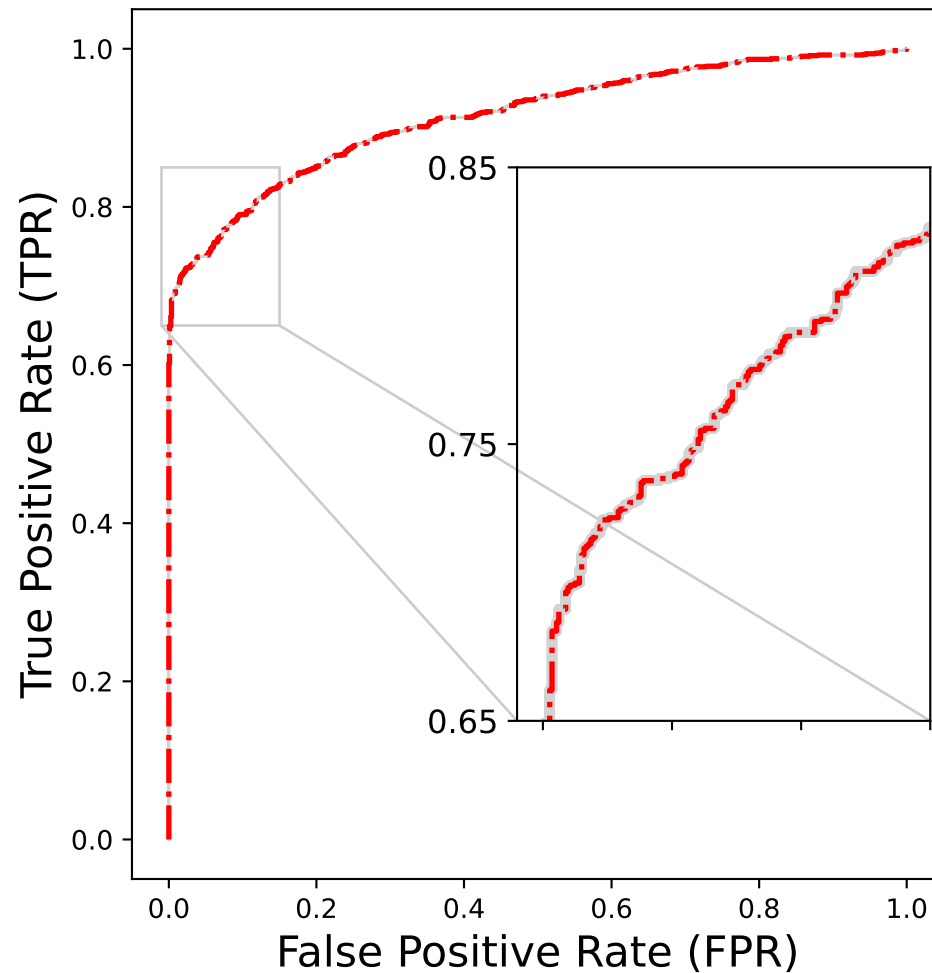

Supplement: Supplementary file 1 [file biology-12-00518-s001.zip › biology-2211468-supplementary/Supplementary/Figure S5.pdf]

# RNA-seq TPM ROC curve

## REWKLR

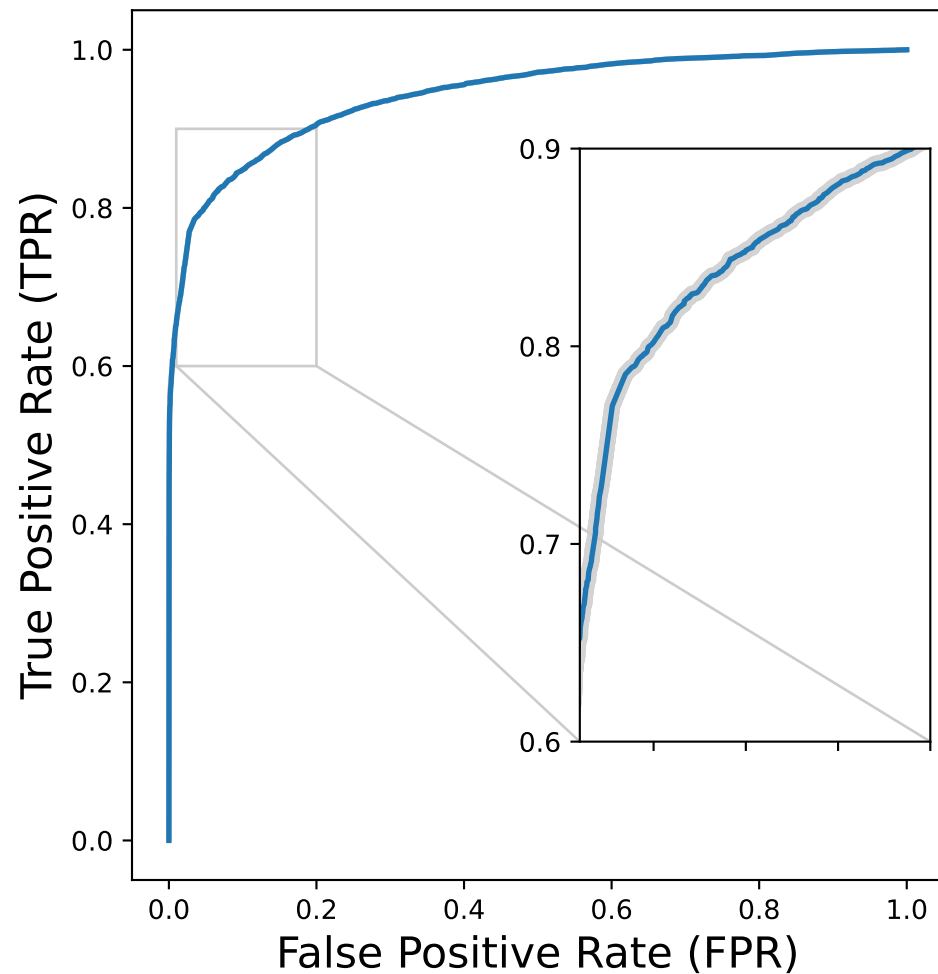

## SVM

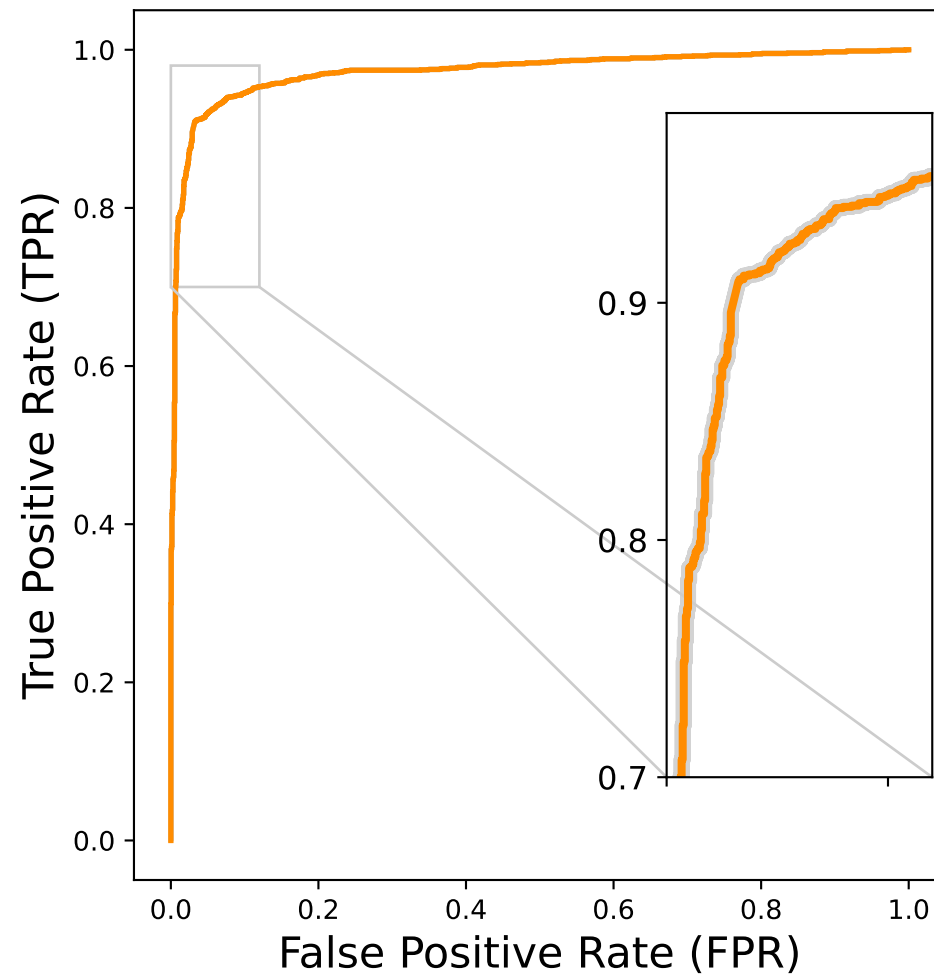

Supplement: Supplementary file 1 [file biology-12-00518-s001.zip › biology-2211468-supplementary/Supplementary/Figure S6.pdf]

# RNA-seq FPKM ROC curve

## REWKLR

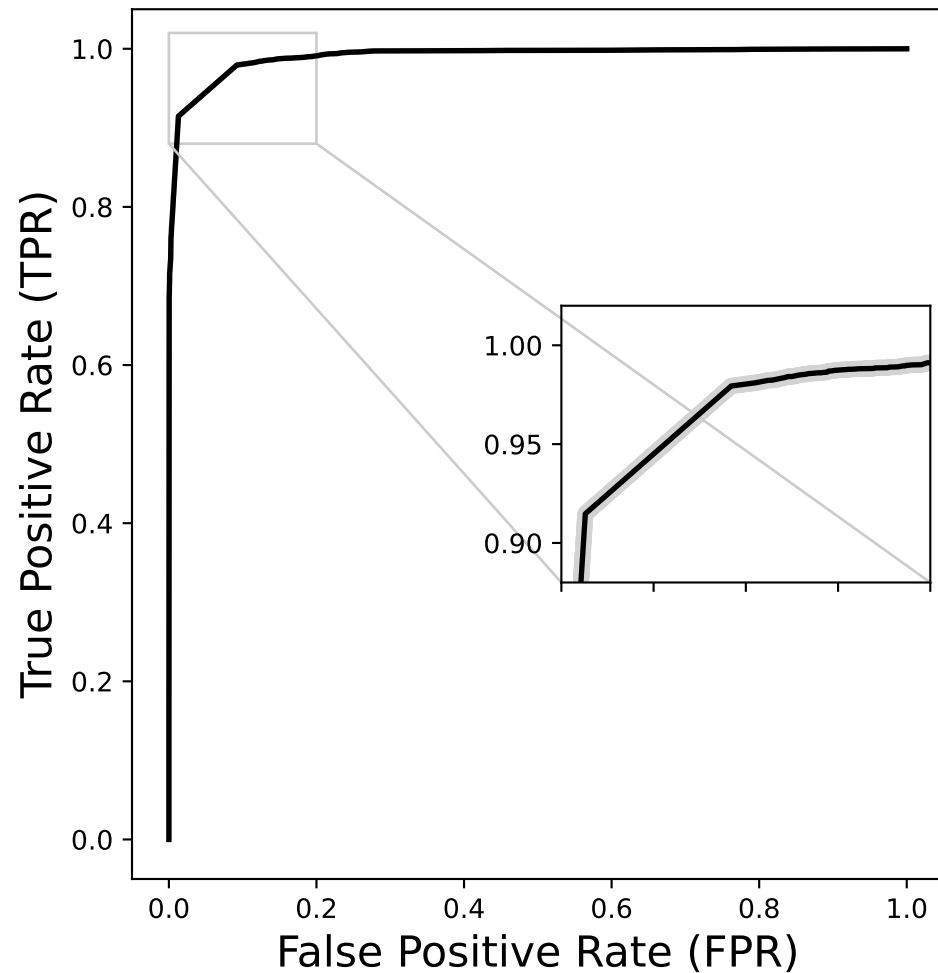

## SVM

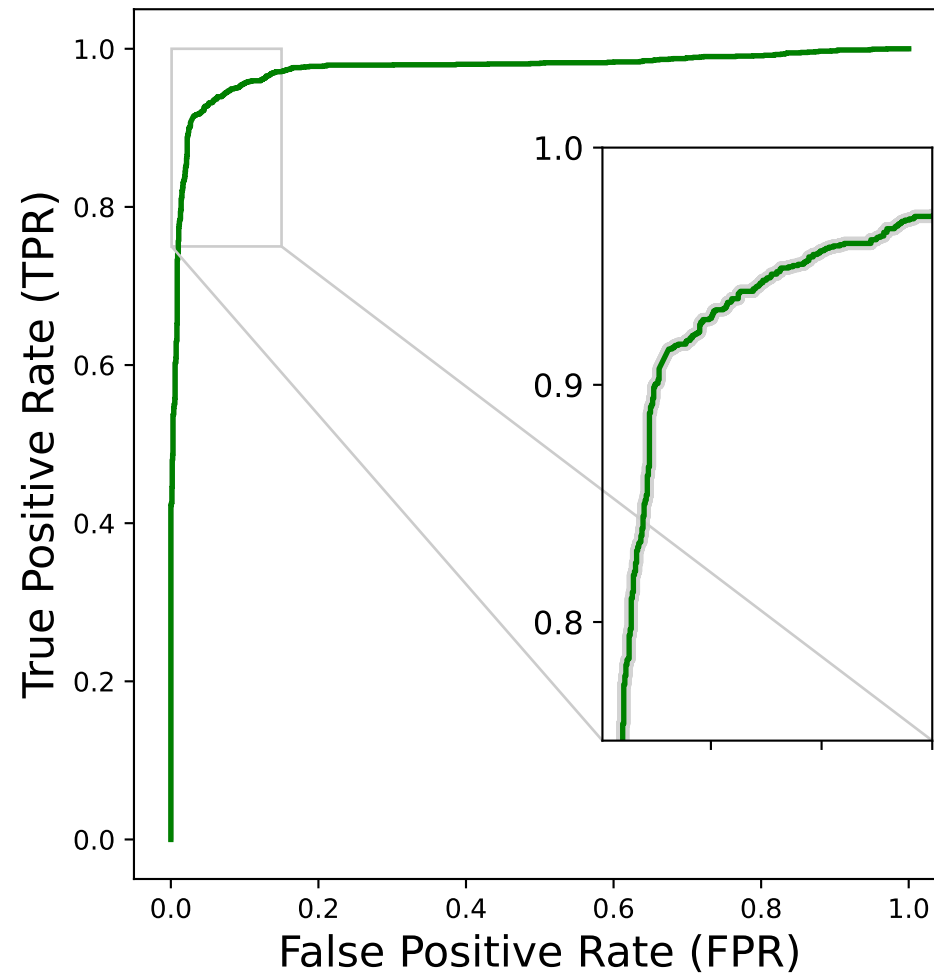

Supplement: Supplementary file 1 [file biology-12-00518-s001.zip › biology-2211468-supplementary/Supplementary/Figure S7.pdf]
